# Genome-Wide Profiling of PARP1 Reveals an Interplay with Gene Regulatory Regions and DNA Methylation

**DOI:** 10.1371/journal.pone.0135410

**Published:** 2015-08-25

**Authors:** Narasimharao Nalabothula, Taha Al-jumaily, Abdallah M. Eteleeb, Robert M. Flight, Shao Xiaorong, Hunter Moseley, Eric C. Rouchka, Yvonne N. Fondufe-Mittendorf

**Affiliations:** 1 Department of Molecular and Cellular Biochemistry, University of Kentucky, Lexington, Kentucky, United States of America; 2 Department of Computer Engineering and Computer Science, University of Louisville, Louisville, Kentucky, United States of America; 3 McDonnell Genome Institute, Washington University in St. Louis, St. Louis, Missouri, United States of America; 4 Division of Epidemiology, School of Public Health, University of California, Berkeley, California, United States of America; National Cancer Institute, UNITED STATES

## Abstract

Poly (ADP-ribose) polymerase-1 (PARP1) is a nuclear enzyme involved in DNA repair, chromatin remodeling and gene expression. PARP1 interactions with chromatin architectural multi-protein complexes (i.e. nucleosomes) alter chromatin structure resulting in changes in gene expression. Chromatin structure impacts gene regulatory processes including transcription, splicing, DNA repair, replication and recombination. It is important to delineate whether PARP1 randomly associates with nucleosomes or is present at specific nucleosome regions throughout the cell genome. We performed genome-wide association studies in breast cancer cell lines to address these questions. Our studies show that PARP1 associates with epigenetic regulatory elements genome-wide, such as active histone marks, CTCF and DNase hypersensitive sites. Additionally, the binding of PARP1 to chromatin genome-wide is mutually exclusive with DNA methylation pattern suggesting a functional interplay between PARP1 and DNA methylation. Indeed, inhibition of PARylation results in genome-wide changes in DNA methylation patterns. Our results suggest that PARP1 controls the fidelity of gene transcription and marks actively transcribed gene regions by selectively binding to transcriptionally active chromatin. These studies provide a platform for developing our understanding of PARP1’s role in gene regulation.

## Introduction

Chromatin, comprising repeating units of nucleosomes, plays a vital role in gene expression by regulating the access of regulatory proteins to their target binding sites. This access is controlled by the locations of nucleosomes along genomic DNA. Interestingly, nucleosome positions are controlled by various factors including DNA sequence [1–4], incorporation of histone variants, histone posttranslational modifications, DNA methylation and chromatin architectural proteins (CAPs) [5]. The complex molecular mechanisms through which chromatin binding proteins, DNA sequence, and histone modifying enzymes coordinate to alter chromatin structure and function to regulate gene expression require more focused study to elucidate their specific interplay.

In this study, we aimed to determine the genome-wide functional location of Poly (ADP-ribose) Ribose Polymerase-1 (PARP1) also known as ADP-ribosyl transferase1 (ARTD1), an NAD-dependent chromatin-associated protein. PARP1 is widely known for its role in DNA repair and cell death [6,7]. Recent studies have now extended the physiological roles of PARP1 to include regulation of gene expression at both the transcriptional and splicing levels [8–10]. These studies suggest that PARP1 affects gene expression via its chromatin remodeling activity [11,12]. Several possible mechanisms exist through which PARP1 could regulate chromatin structure. First PARP1 binds at the entry/exit sites between the nucleosome and linker DNA [13] and may influence spontaneous nucleosome opening [14,15] thereby allowing access to DNA by regulatory proteins [16–18]. Second, as a chromatin remodeler, PARP1 PARylates core histones, linker histone H1 and chromatin architectural proteins (CAPs) such as histone H1 and HMGN1 [19–24]. Third, PARP1 competes with histone H1 for binding to nucleosomes [25]. All of these activities result in changes to chromatin structure and gene regulation. Although PARP1 undergoes auto-PARylation during transcriptional activation, several transcription repressor complexes undergo PARylation followed by dissociation from gene promoters [26], resulting in gene repression. These studies therefore implicate PARP1 both in transcriptional repression and activation.

Indeed PARP1 is found both at silenced [27] and activated genomic loci [25,28,29]. PARP1 modifies histones, recruits and modulates the activity of histone variants [22,27,28] and remodelers [21], and disrupts nucleosomes [24,30,31]. To begin to decipher the mode of action of PARP1 in transcription regulation, it is imperative to profile PARP1’s functional genome-wide location. In this study we carried out nucleosome ChIP-seq experiments to determine the functional location of PARP1-bound nucleosomes in human cells. We found that PARP1 is associated with active histone modifications and is bound to regulatory regions. PARP1’s role in gene regulation may be explained through an active competition of PARP1 chromatin binding and DNA methylation. We performed additional genome-wide methylation analyses, which showed changes in methylation patterns resulting from inhibition of PARylation. These studies demonstrate an intricate reciprocal interplay between PARP1 and DNA methylation, providing a platform to delineate PARP1’s functional role. We explore this role in two cancerous model cell lines that respond differently to PARP1 inhibitors.

## Materials and Methods

### Cell culture and nuclei isolation

MDA-MB231 and MCF7 cells were cultured in DMEM (Invitrogen) supplemented with 10% FBS (Hyclone). 100 x 10^6^ cells were cross-linked with 1% formaldehyde at RT for 10 min. The cross linking reaction was stopped with 125 mM glycine. Cells were then lysed in NP-40 hypotonic lysis buffer (10 mM Tris [pH 7.4], 10 mM NaCl, 3 mM MgCl_2_, 0.5% NP-40, 0.15 mM spermine, 0.5 mM spermidine, complete protease inhibitor cocktail) for 30 minutes followed by dounce homogenization.

#### SiRNA treatment of cells

siRNA targeting the coding sequence of β-galactosidase (LacZ) was used as a non-specific control. SiRNAs against human PARP1 (ON-TARGET*plus*) from Thermo Scientific Dharmacon and transfection was performed as per the manufacturer using Dharmafect reagent 2.

Treatment of cells with Aza-cytidine: Cells were treated with 1 μM aza-cytidine for 2 days. Water treated growth match controls were used. In all the experiments, cell viability was determined using trypan blue exclusion test while apoptosis was measured with the Annexin V-PI kit according to the manufacturer's protocol (Trevigen).

#### Treatment of cells with PJ34 treatment

Cells were treated with PJ34 similar to Krishnakumar et al, 2010 [29], with slight modification–to ensure complete effect of PARylation inhibition, cells were treated with 5 μM PJ34 overnight (~16 hours).

#### Treatment of cells DPQ

MCF7 cells at 70% confluency were treated with 10 μM 3,4-Dihydro-5[4-(1-piperindinyl0butoxy]-1(2H)-isoquinoline (DPQ) or overnight for about 16 hours, an equal volume of DMSO was used for control.

### Nucleosome-Chromatin Immunoprecipitation (nuc-ChIP)

ChIP was performed as described by [32,33] with minor modifications. Chromatin was cross-linked with 1% final formaldehyde concentration for 8 min at room temperature. Cross-linking was stopped by a final concentration of 0.125 M glycine incubated for 5 min at room temperature on a rocking platform. The medium was removed and the cells were washed twice with ice-cold PBS. Cells were lysed by incubating for 5 mins in cold lysis buffer (1% SDS, 10 mM EDTA, protease inhibitors, 50 mM Tris–HCl, [pH 8]). Resulting nuclei were washed in MNase buffer (10mM Tris [pH 7.4], 15mM NaCl, 60 mM KCl, 0.15 mM spermine, 0.5 mM spermidine, 2 mM CaCl_2_). Chromatin was subjected to micrococcal nuclease (MNase) digestion, with pre-determined MNase concentration, in MNase buffer at RT to yield nucleosomal fragments with less than 3 nucleosomes (~450 bp). 25 mM EDTA and 0.2% SDS was used to stop the reaction and sample was centrifuged to remove cellular debris. Lysates were diluted 1:10 in ChIP dilution buffer (0.01% SDS, 1.1% Triton X-100, 1.2 mM EDTA, 167 mM NaCl, protease inhibitors, 16.7 mM Tris–HCl, [pH 8]). Non-specific background was removed by incubating the MNase digested chromatin with dynabeads (Invitrogen) overnight at 4°C with rotation in the presence of BSA (250 μg/ml). Precleared chromatin solutions were incubated overnight at 4°C with rotation with antibodies against PARP1 (#39559, Active Motif) and for control, IgG. The immuno-complexes were collected with 200 μl of MagnaChIP protein A beads (Millipore) for 3 h at 4°C with rotation. The beads were washed sequentially for 3 min by rotation with 1 ml of the following buffers: low salt wash buffer (0.1% SDS, 1% Triton X-100, 2 mM EDTA, 150 mM NaCl, 20 mM Tris–HCl, [pH 8]), high salt wash buffer (0.1% SDS, 1% Triton X-100, 2 mM EDTA, 500 mM NaCl, 20 mM Tris–HCl, pH 8.1) and LiCl wash buffer (0.25 M LiCl, 1% Nonidet P-40, 1% sodium deoxycholate, 1 mM EDTA, 10 mM Tris–HCl, [pH 8]). Finally, the beads were washed twice with 1 ml TE buffer (1 mM EDTA, 10 mM Tris–HCl, [pH 8]). The immuno-complexes were then eluted by adding 500 μl of elution buffer (25 mM Tris–HCl, [pH 7.5], 10 mM EDTA, 0.5% SDS) and incubating for 60 min on rotation. The cross-linking was reversed at 65°C and the remaining proteins were digested with 80 μg/ml proteinase K (Fermentas) and incubating overnight at 65°C. The DNA was recovered by purifying with Qiagen PCR purification kit. Immunoprecipitated chromatin DNA was then run on a 3.3% Nusieve gel. Mononucleosomal fragments were cut from the gel and adapted for deep-sequencing as we described previously [34]. For ChIP-qPCR experiments, eluted samples were used for quantitative real-time PCR or normal PCR. For MNase-seq analyses, experiments were performed as we had previously done [35].

### Preparation of ChIP-seq libraries

Sequencing libraries were prepared with the SOLiD ABI technologies as previously described [36]. Briefly: ChIPped mononucleosomal DNA was gel purified, ligated with the adapters and then PCR amplified with SOLiD primers for 10 cycles. PCR product, corresponding to mono-nucleosomal DNA, was gel purified and loaded onto a SOLiD flow cell for cluster generation and genome-wide sequencing.

### Data Analyses

PARP1 colorspace fasta reads were converted to basespace fastq format by the Northwestern University Genomics core using a custom script. We sorted PARP1 reads into separate library files based on their barcodes, and mapped the reads to the human genome (hg19) using bowtie v2 with the default parameters allowing for two mismatches [37]. Unique reads with zero or one mismatch were considered for further analysis. Amplification bias was removed by filtering more than two copies of redundant reads that come from the same locus. We compared how well technical replicates within each cell of PARP1 binding overlapped each other. The degree of overlap among peak-sets for all two biological replicates was more than 75%, demonstrating a high level of reproducibility (**S1A Fig**) further confirmed with a Pearson correlation of r > 0.823; p< 10−^16^ (**S1B Fig**). Because of this high reproducibility between the replicates, the unique reads were combined and reanalyzed to produce composite binding regions. A total of 54,125,493 and 60,486,178 non-redundant uniquely mapped reads were obtained for MCF7 and MDA-MB231 cells respectively.

#### Defining PARP1 binding sites

Two different methods were used to analyze PARP1 binding regions since no peak-calling program has gained consensus acceptance by the scientific community as the preferred tool for ChIP-Seq data analysis [38].

First method: The mean nucleosomal fragment length of each library was estimated by computing the offset yielding the highest covariation of read depth between the forward and reverse strands. The PARP1 nucleosome midpoint locations were estimated as the read start plus the offset (or minus the offset for those mapping to the reverse strand). Next we estimated the distribution of fragment sizes from separation of read pairs, and discarded read pairs outside of the central 95% of distribution (101–191 bp). Finally we estimated nucleosome midpoints as the midpoint between read pairs.

Second method: MACS (Model-based Analysis for ChIP-Seq) was used to identify peaks from the ChIP-Seq data and therefore determine PARP1 binding sites. For each replicate, peaks were identified with a default p-value significance of 1x10^-5^. ChIP-Seq peaks were detected using Macs2 [39,40] with the broad option and a window size of 200 bp. Overlaps between each ChIP-Seq data peaks (CTCF, histone modifications and PARP1 replica) and PARP1 peaks were determined by using an overlap of at least 10% of the ChIP-Seq peak with the PARP1 peak. MNase-seq data was used as ‘input’ control in calling PARP1-peaks.

Both methods yielded very similar results, while having different limitations: method 1 produces background noise and method 2 possibly eliminated PARP1 binding sites,.

In order to visualize PARP1-bound nucleosome tag-density across the human chromosomes on the UCSC browser for hg19, ready-to-visualize bedgraph files were created using the HOMER package v3.13 [41]. Briefly, aligned reads were extended to the average fragment size (~160 bp) and read coverage on each base across the genome was calculated. Read coverage was then scaled to one million and normalized with the total number of reads. All publicly available data used for pairwise comparisons with our PARP1 data were processed in the same way. We also used total nucleosome (nucleosome-seq) data to normalize for background correction.

We conducted a Gene Ontology analysis using the Database for Annotation, Visualization, and Integrated Discovery (DAVID) [42] on genes with a PARP1 peak within 1 kb of the TSS as determined by MACS (P < 10^−5^) or by our method.

Correlations with DHS and CTCF sites: A list of previously identified CTCF sites for MCF7 cells [43,44] was obtained (UCSC ModEncode database), and the correlations of PARP1 and CTCF binding was calculated using a custom script (figshare [45]). A Similar procedure was applied to align PARP1 ChIP-Seq data with DNase hypersensitive sites (DHS) using DHS data from UCSC genome database [46,47]. These analyses were performed at TSSs as well as in 2 kb windows across the genome, providing genome-wide information.

Correlations with histone modifications: ChIP-Seq data for the various histone modifications in MCF-7 cells were downloaded from the UCSC ENCODE data portal [48]. We took 2 kb windows surrounding all annotated TSSs and computed mean values for each histone modification experiment in each window. For the same windows, we also computed normalized values for the PARP1 experiments by dividing the number of midpoints from a given experiment by the number total nucleosome midpoints and taking the log. We then computed Pearson *R* correlations across windows for all possible pairs of experiments. Similarly we divided the genome into 2 kb windows and measured the correlation. Custom script is available in figshare [45,49–51].

Mapping PARP1 signals across high and low expressed gene TSS: PARP1 ChIP-Seq tags from both cell lines were aligned with TSS coordinates from Ensembl transcript of high and low expressed gene RNA-Seq data [52]. PARP1 nucleosomal reads were mapped to +/- 1000 bp surrounding known TSS using a 5 bp sliding window. These 2 kb windows were generated across the human genome (genome wide) using the *tileGenome* function from the *GenomicRanges* package [53] and the *BSgenome*.*Hsapiens*.*UCSC*.*hg19* package (BSgenome.Hsapiens.UCSC.hg19 1.3.1). Mapped reads were normalized to total reads in each ChIP-Seq data set. Since TSS data has a strand option, end number position was used for negative strand, whereas positive number position was used for a positive strand to match against the same strand and chromosome from our ChIP-Seq libraries. We computed the Pearson correlation between gene expression and the density of PARP1 and nucleosome midpoints (center) density in each region.

### Real-Time qPCR (qPCR)

ChIP was performed on MNase digested chromatin fragments as before using anti-PARP1 antibodies. 6 μg of antibody, 100 μl of dyna beads and 400 μg of chromatin were used for each ChIP. IgG was used as a no-antibody control. Antibody bound (IP) chromatin DNA along with no-antibody control was analyzed by qPCR using the PerfeCTa SYBR Green FastMix (Quanta Biosciences Inc, USA) and C1000 Thermal Cycler (Bio-Rad, USA) following the manufacturer's instructions. The primers used to amplify the selected gene promoters were as follows: IGFBP6: Forward 5’-ccaccacaatgctagggtct-3’, Reverse 5’-cagactggggtaagccaatg-3’; IGFBP7: Forward 5’-ggcaacggtttgtcctttta-3’, Reverse 5’-aaaacccccacttgtggact-3’. 20 μl reactions was set up using 1 μl of ChIP DNA, 10 μl of 2x PerfeCTa SYBR Green FastMix and 0.5 μM each of gene specific primers. The cycling conditions were: 95°C for 2:00 m; 40 cycles of 95°C for 30 s, 55°C for 30 s and 72°C for 1:00 m. Fluorescence was measured right after each elongation step. Dissociation curves were used to confirm specificity of PCR products. “Signal over background” normalization method was used to calculate fold enrichment [54].

### Analysis of PARP1 functional binding at homo- and hemi-methylated CpG and non CpG sites

Genome-wide methylation profiles of CpG and non-CpG (CWG) sites in MDA-MB231 cells were obtained from NGSmethDB [55] database. The total number of methylated reads at each location was calculated as the number of aligned reads multiplied by the percentage of those reads that were methylated. This total number of methylated reads was summed for each 2 kb window. The binding profiles of PARP1 at methyl CpG and CWG sites were created by aligning the above genome-wide methylation profiles with PARP1 ChIP-Seq libraries. The ratio of the number of PARP1 ChIP-Seq reads found in a 2 kb region centered on the methyl CpG or CWG site over the mean number of PARP1 ChIP-Seq reads found throughout the genome were plotted. A similar procedure was used to align histone modification profiles with PARP1 ChIP-Seq libraries using genome-wide histone modification date generated [56,57].

DNA sample preparation, bisulfite conversion and methylation level measurement: DNA was extracted from experimental samples using the Genomic DNeasy kit (Qiagen), as described by the manufacturer. DNA quantification was performed using a nanodrop. 750 ng of the DNA was used as input for the bisulfite conversion using the Zymo EZ-96DNA Methylation Kit (Catalog #D5004) Deep-Well Format. Then 4 ul of the bisulfite converted DNA was used as input for the Illumina Infinium HD Methylation Assay and performed per Illumina’s standard protocol. Processed methylation chips were scanned using an iScan reader (Illumina). Paired samples (vehicle-treated and corresponding PJ34-treated samples) were processed on the same chip, and all samples were processed at the same time to avoid chip-to-chip variation.

Data quality control and preprocessing: Infinium methylation data were processed using the Methylation Module of the GenomeStudio software package (v. 2011.1). Target success rate was determined. The detection p-value is the 1-p computed from the background model characterizing the chance that the target sequence was distinguishable from the negative controls. For quality control, methylation measures with a detection p-value >0.05 and samples with a CpG coverage <95% were removed. Ultimately all samples passed the coverage criteria. The data were initially normalized using internal controls in the GenomeStudio software. Sample replicates and Jurkat control replicates were checked to ensure an r^2^ value >0.99.

Determination of differential methylation patterns: The methylation levels of CpG sites were calculated as β-values (β = intensity (methylated) / intensity (methylated + unmethylated)). A non-normal distribution of DNA methylation within each sample was observed. Therefore, a non-parametric method, paired two-sample Wilcoxon signed-rank test was performed to assess the differences in global DNA methylation between the treated and non-treated samples. To assess the difference of DNA methylation level between the treated and untreated samples at a single CpG site, the absolute value of the log_2_ fold change was calculated. CpGs are considered differentially methylated if the absolute value of the log_2_ fold change is greater than one. A hypermethylated CpG site is observed if the DNA methylation level is higher in the treated sample; a hypomethylated CpG site is observed if the DNA methylation level is higher in the untreated sample. The data were further normalized using a peak correction algorithm embedded in the IMA-R package.

DNA isolation and MSRE-PCR: Genomic DNA was isolated from cells using DNeasy kit (Qiagen). About 1 μg of genomic DNA was digested at 37°C for 8 hours with 20U of *HindIII* and further digested with overnight with 20U of methylation sensitive enzymes *HhaI* and *HpaII* (NEB). All restriction enzymes were from New England Biolabs. DNA was purified using Qiagen PCR purification kit and used for PCR analyses. PCR amplification was performed using Taq DNA polymerase (Invitrogen). The PCR conditions were 95°C for 5 min followed by 35 cycles at 95°C for 45 s, 60°C for 45 s, 72°C for 1 min and a final extension of 72°C for 10 min. MethPrimer [58] was used for primer design; primer sequences are available upon request.

## Results

### PARP1 binds to promoters of active genes

To determine the genome-wide location of PARP1, we carried out nucleosome-chromatin immunoprecipitation followed by deep-sequencing (nuc-ChIP-seq) in the human cell lines MCF7 and MD-MB231 [59]. Following nucleosome-ChIP analysis, the DNA fraction of the eluted ChIP sample was run on a 3.3% nusieve agarose gel (**S2A Fig**). Western blot analyses confirmed the eluted fraction contained PARP1 bound nucleosomal fragments (**S2B Fig**). The mononucleosomal DNA fragment was then excised from agarose gel and subjected to paired-end deep-sequencing. After sequencing, we aligned the reads to the human genome (HuRef-Hg19) and discarded non-unique reads. Two to three replicas that showed reproducibility from each cell line were used to build a composite binding data set for PARP1. A total of 5–6 X 10^7^ paired-end reads were retained for PARP1-bound nucleosomes in MCF7 and MDA-MB231 cells, respectively. This corresponds to ~4 fold coverage if every nucleosome is PARP1-bound. However, since PARP1 binds selectively, we believe the coverage to be even higher. We then used these reads to determine the functional location of PARP1-bound nucleosomes. The number of PARP1-ChIP-seq nucleosome centers within each region was counted and divided by the total number of mapped reads to estimate the relative abundance of PARP1-bound nucleosomes per genomic region.

**Fig 1:**
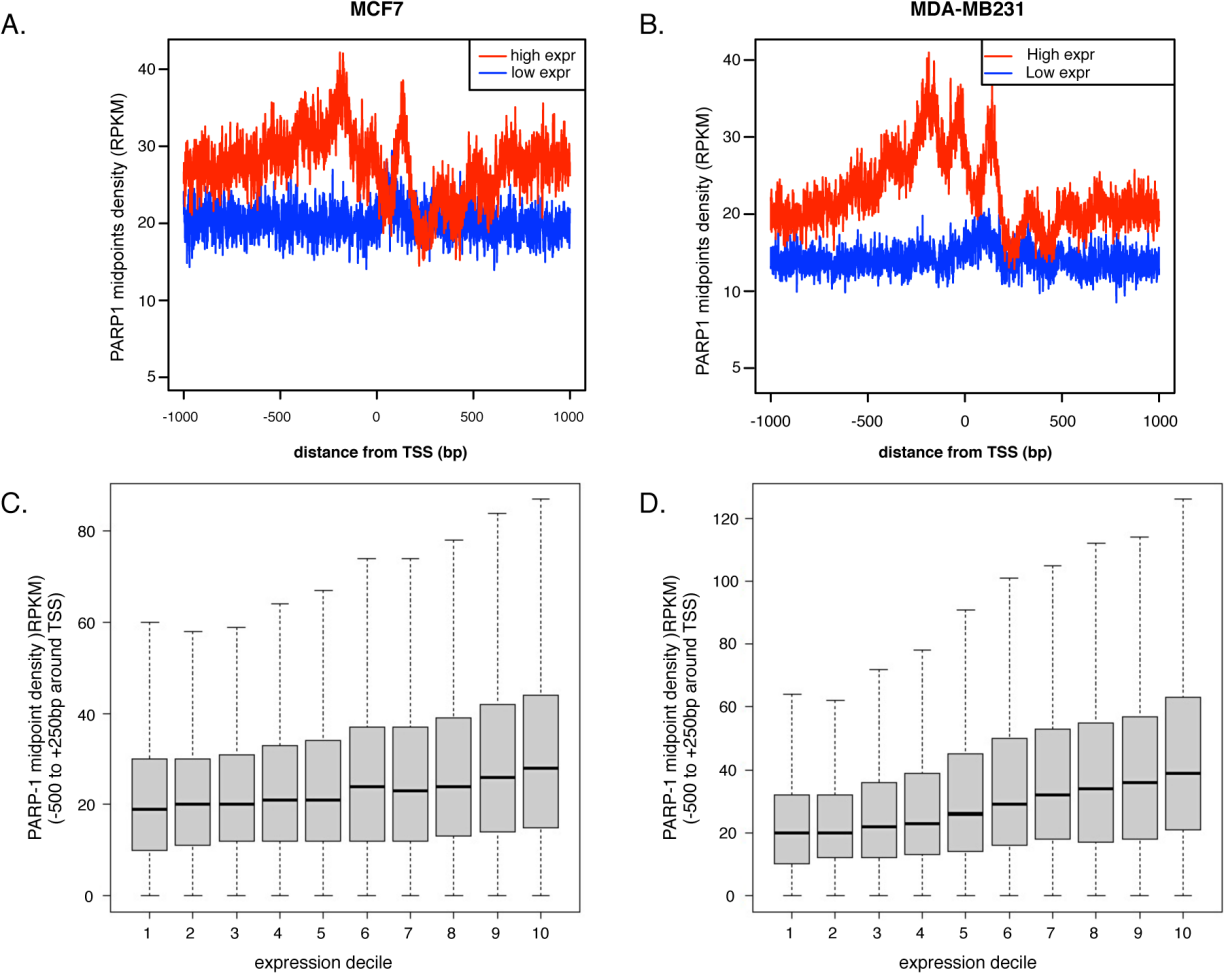
PARP-1 binds promoters of active genes. Midpoints of the reads of PARP1-associated nucleosomes were plotted against distance from TSSs. PARP-1 associates with highly transcribed gene TSS’s (solid red line), and not with promoters of low expressed or repressed gene promoters (solid blue) in (A) MCF7 cells and (B) MDA-MB231 cells. All genes were ranked by level of expression in both MCF7 and MDA-MB231 cells and sorted into deciles from lowest to highest. Mapping of PARP1 association show increased PARP-1 enrichment at TSS as level of gene expression increases in (C) MCF7 and (D) MDA-MB231 cells.

To determine the functional binding of PARP1 genome-wide, we analyzed its binding to key gene regulatory regions. We showed recently in *Drosophila* cells that PARP1 binds to active promoters (Matveeva et al., 2015—submitted), corroborating earlier lower resolution data in human MCF7 [25]. We therefore asked whether this is true in human cell lines using the higher resolution analyses obtained with nuc-ChIP-seq. For this, we assayed for PARP1-nucleosome binding sites within 1 kb of transcription start sites (TSSs). Only genes with some evidence for expression (>0 RNA-seq signal) were used. We called genes low or high expression if they fell into the lower or upper quartiles of these genes. In total, 4985 low expression TSSs and 5196 high expression TSSs were used. Generally, PARP1 occupied promoters of highly expressed genes (upper quartile of expressed genes) and was absent at the promoters of repressed genes (bottom quartile of expressed genes) both in MCF7 (**Fig 1A**) and MDA-MB231 cells (**Fig 1B**). To further test the correlation between PARP1 binding and gene expression, we divided gene activity into 10 deciles based on the level of gene expression. For this analysis we counted the number of midpoints in the region from -500 to +250 around the TSSs. Here too, we observed that the higher the gene expression levels, the more binding of PARP1 to the promoters of these genes (**Fig 1C and 1D**), suggestive of an association of PARP1 with gene activity.

We tested and validated our results from nuc-PARP1-ChIP-seq using ChIP followed by quantitative real time PCR at two PARP1-binding representative genes. When comparing the IGFBP7 and IGFBP6 gene promoters in MDA-MB231 cells to MCF7 cells, we observed higher PARP1 occupancy signals (**S3A and S3B Fig**). To validate the binding of PARP1 to active promoters, we measured the relative PARP1 occupancy at active promoters in the two cell types. The ChIP-qPCR results confirm our nuc-ChIP-seq data (**S3C and S3D Fig).** Interestingly, IGFBP7 and IGFBP6 are both expressed in MDA-MB231 cells and not in MCF7 cells [60–62] (**S3E Fig**). Additionally knockdown of PARP1 (**S4A and S4B Fig**), showed a depletion of PARP1 occupancy at 3 PARP1-target sites as measured by qPCR (**S4C and S4D Fig**). Together, our results show a positive correlation between PARP1 enrichment at promoter regions and the level of gene expression: the higher the gene expression the more the enrichment of PARP1. These observations are in line with lower resolution studies that show PARP1 binds to active gene promoters [24,25].

### PARP1 associates with distinct histone marks and DNase I hypersensitive sites

**Fig 2:**
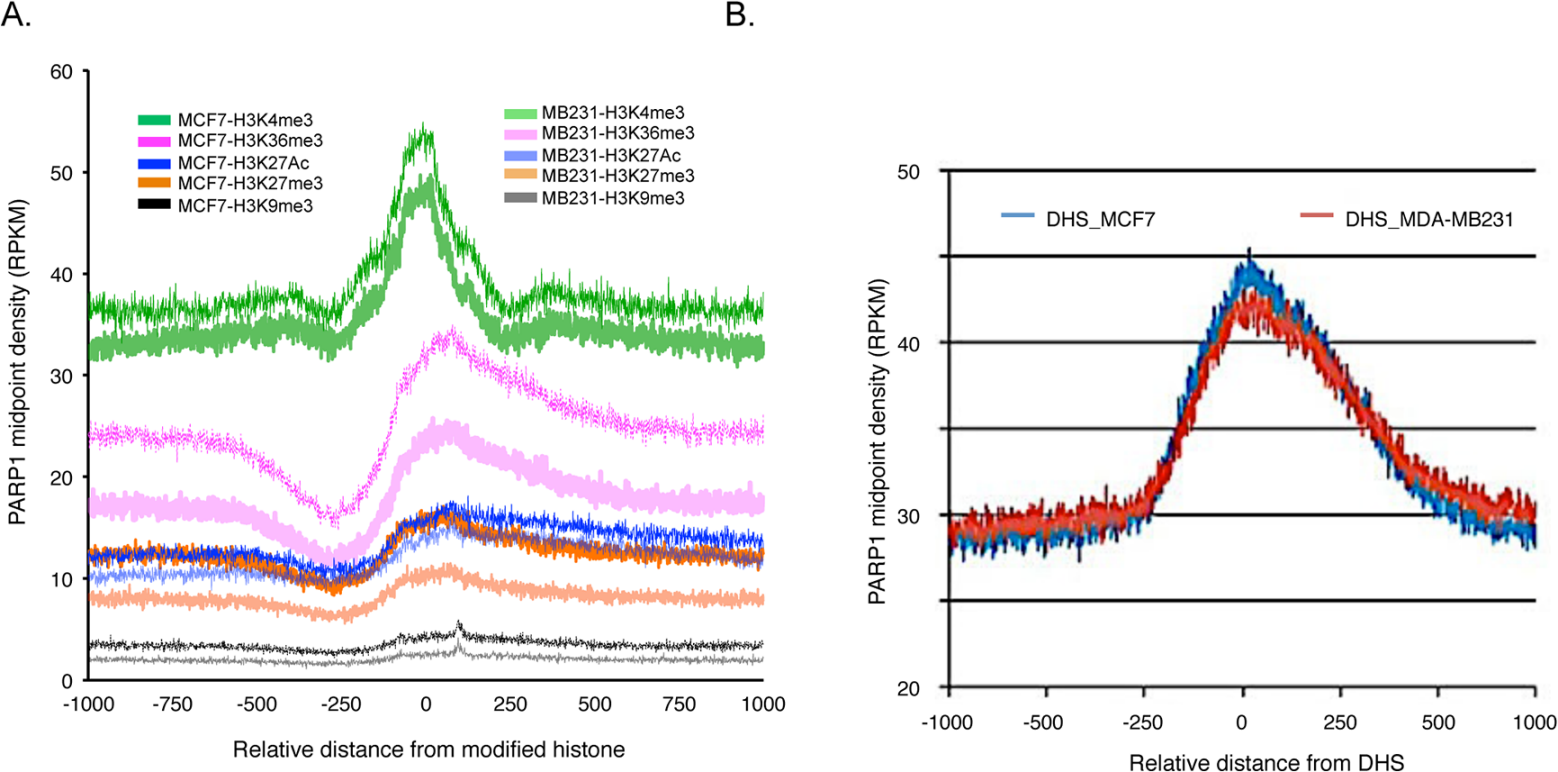
PARP-1 is enriched at active epigenetic marks. (A) PARP1 associates with genomic regions containing specific histone modifications. Genomic locations of various histone modifications were mapped on PARP-1 bound regions of the human genome. Solid green, red, blue, orange and black lines represent PARP-1 alignment with H3K4me3, H3K36me3, H3K27ac, H3K27me3, and H3K9me3 modifications in MCF7 and MDA-MB231 cells. (B) PARP1 also binds to DNase hypersensitive sites in both cell types.

Gene expression is associated with the presence of different types of histone marks present at the promoters of genes. H3K36me3, H3K4me3, and H3K27ac are considered “active” marks, whereas H3K9me3 and H3K27me3 are considered “repressive” marks [63]. We therefore asked whether the binding of PARP1 correlated with the presence of particular histone marks. ChIP-seq reads of various histone marks were obtained [48] and used to correlate the binding of PARP1 to genomic regions containing these histone marks. Using 2 kb windows around annotated TSSs, we computed the mean signal for each histone PTM and estimated the density of PARP1-bound nucleosomal reads. We found that PARP1 associated with the activating histone PTM H3K4me3, and with the elongating mark H3K36me3 (**Fig 2A**). On the other hand, PARP1 associated neither with regions containing the activating mark H3K27ac nor with the repressive heterochromatin marks H3K9me3 and H3K27me3. We further quantified the correlation of PARP1 and histone PTMs genome-wide (**S1 Table**) and observed a moderate positive Pearson correlation with H3K4me3 at TSSs of r = 0.357 (*p = 0*.*4e-16*) and r = 0.3447 (*p < 0*.*5e-16*) in MCF7 and MDA-MB231 cells respectively. However, PARP1’s correlation with other PTMs varied from no relationship to moderate negative correlation and differed across cell lines (figshare [50]).

DNase 1 hypersensitive sites (DHSs) mark regulatory regions on DNA such as enhancers, promoters, silencers, insulators and locus control [64]. In order to further define PARP1’s role in gene regulation, we mapped the association of PARP1 binding to DHSs. DHS data in MCF7 and MDA-MB231 cells were obtained from the UCSC Genome Browser [46,47] and used to correlate the relative enrichment of PARP1 within 1 kb regions surrounding the DHSs. We found that the density of PARP1 nucleosome midpoints is much higher near DHSs (**Fig 2B**) in both cell types.

### PARP1 co-localizes with nucleosomes positioned at CTCF flanking regions

**Fig 3:**
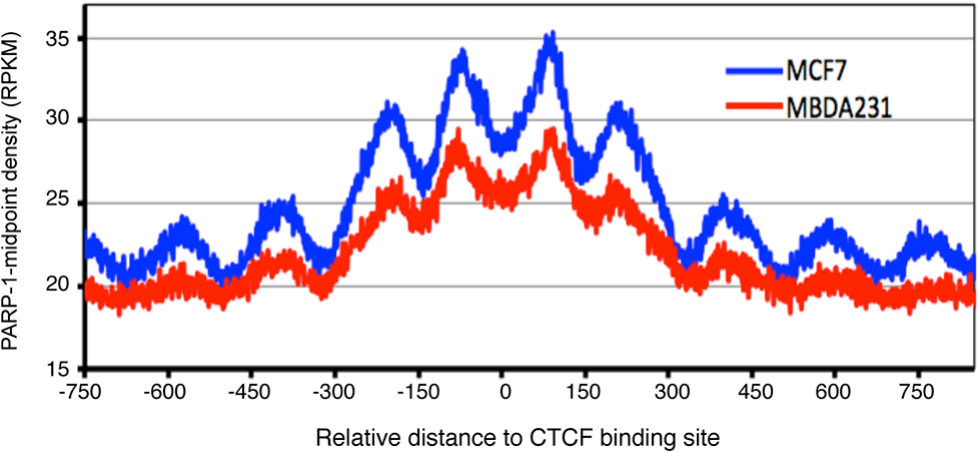
CTCF flanking regions overlap with PARP-1-associated nucleosomes. CTCF binding sites on genome scale were mapped with genome-wide PARP-1 associated regions in breast cancer cells in MCF7 cells (blue) and MDA-MB231 cells (red). We quantified the correlation between PARP1 binding and CTCF binding and showed a Pearson correlation r of about 0.18 (p < 0.05) in both cell types (figshare) [50]).

Chromatin organization into distinct functional domains is important for the temporal and spatial gene expression patterns required for proper development in the mammalian genome [65]. One such chromatin organizer is CCCTC-binding factor (CTCF), a sequence-specific transcription factor that binds to its target site and links chromosomal domains [66]. Thus CTCF binding sites relate to gene regulatory regions. Indeed, earlier genome-wide analysis studies identified well-positioned nucleosomes and DNase 1 hypersensitive sites as flanking CTCF-binding sites [67]. Additionally, CTCF directly induces PARP1’s PARylation activity in the absence of DNA damage, suggestive of an interaction between these two proteins *in vivo* [68]. Thus, to further explore the functional binding of PARP1, we performed a genome-wide characterization of its binding to CTCF binding sites. For this, we measured CTCF binding sites in MCF7 cells [69] and used these to analyze PARP1 binding sites in both cell types. We show that PARP1 binds to the nucleosomes flanking the CTCF binding sites in MCF7 cells with Pearson correlation r = 0.2; p < 0.05 (**Fig 3**). We next asked if that is true for MDA-MB231 as well. We simulated the CTCF binding sites from MCF7 onto MDA-MB231 cells. This was done with the assumption that CTCF binding sites are on DNA sequences which do not change from cell type to cell type, though mindful of the fact that ~20% of CTCF binding sites are cell-type specific [70]. Results from MDA-MB231 cells (Pearson correlation r = 0.17; p < 0.05) recapitulated the results from MCF7 cells (**Fig 3**). Since, CTCF PARylation protects certain CTCF regions from DNA methylation, underlining the importance of the cross talk between PARP1, CTCF and DNA methylation in the maintenance of nuclear organization [71]. In line with these studies, we next aimed to determine the relationship of PARP1 binding with DNA methylation status.

### PARP1 binds to hypo-methylated DNA regions

**Fig 4:**
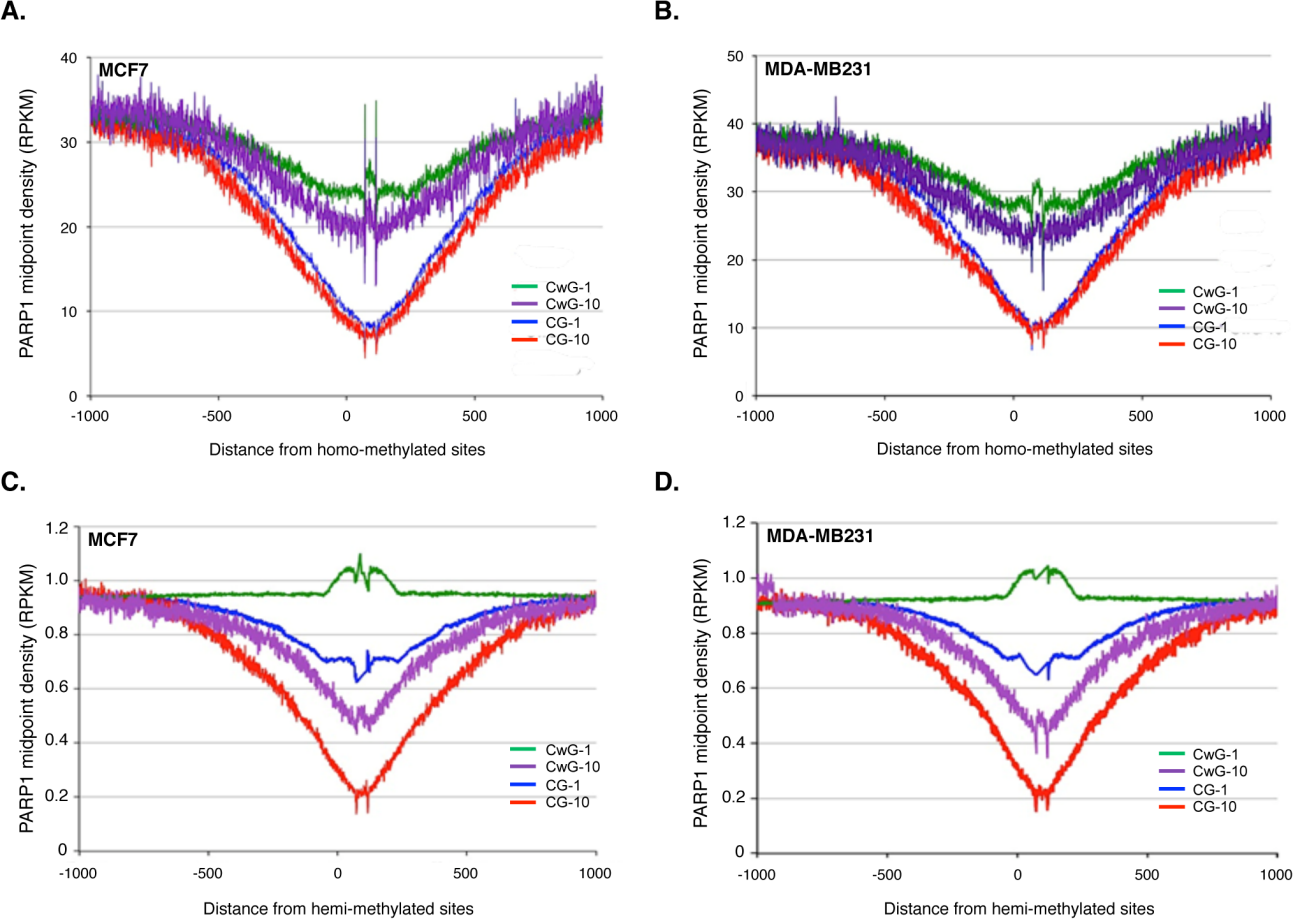
Global analysis of PARP-1 association with methylated DNA. Genome-wide methylated CpG and non-CpG sites were aligned with PARP-1-associated nucleosomal DNA sequences obtained from PARP1-nuc-ChIP-seq and showed mutually exclusive pattern between both signals. (A) PARP1-binding at methylated sites of MCF7 cells (B) MDA-MB231 cells. CG-1 and CG-10 (blue and red) lines are PARP-1 association with moderately and highly methylated sites respectively. CpG sites; CwG-1 and CwG-10 (green and violet) represent PARP1 presence at moderately and highly methylated non-CpG sites (see methods). (C) PARP1 presence at hemi-methylated DNA in MCF7 cells and (D) in MDA-MB231 cells. CG-1 and CG-10 (blue and red lines) indicate moderately and highly hemi-methylated sites while CwG-1 and CwG10 represent moderately and highly hemi-methylated non-CpG sites respectively.

DNA methylation is one of several epigenetic mechanisms that cells use to control gene expression. Several studies show that methylation near gene promoters correlates with gene expression with more methylation of promoters correlating with low or no transcription [72]. Although earlier studies have implicated PARP1’s PARylation of DNA methyltransferase 1 (DNMT1) in DNA demethylation, the functional association and gene regulatory function remains poorly understood [71,73–75]. In an attempt to understand the interplay between PARP1 and DNA methylation, we obtained genome-wide data of CpG methylation in both cell types (GEO: GSM999373 for MCF7 and GSM699947 for MDA-MB231) and divided the methylation status into highly methylated (>10 reads) and less methylated (<10 reads). We then mapped the presence of PARP1 within 250 bp regions of these methylation sites. Our results show that PARP1 binding negatively correlates with the presence and completeness of DNA methylation (**Fig 4A**)—the more methylated the region (>10), the less PARP1 binding was observed at these regions. Conversely, the lower the level of methylation, the more PARP1 enrichment is observed (**Fig 4A and 4B**). While in mammals cytosines are predominantly methylated in the context of the CpG dinucleotide, human cytosine methyltransferases also recognizes CWG and CCWGG (where W stands for A or T) albeit at very low frequency [76]. We therefore analyzed the enrichment of PARP1 at these CWG regions. We divided the methylation status of these CWGs as before, with highly methylated regions being >10 reads and less methylated being <10 reads. Here too, we observed that as the level of methylation decreased, the amount of PARP1 binding increased (**Fig 4A and 4B**). The pattern followed from highest to least PARP1 binding e.g. CWG1>CWG10>CG1>CG10.

The above analyses were done when both strands were methylated. We further tested the enrichment of PARP1 when only one DNA strand is methylated (hemimethylation). Our analyses at hemimethylated sites showed more PARP1 binding at hemimethylated sites compared to homomethylated sites. These results are consistent with mutually exclusive binding of PARP1 and DNA methylation status. Overall, the pattern of PARP1 binding from most to least binding is as follows: hemi-CWG1 > hemi-CpG1 > hemi-CWG10 > hemi-CpG10 > homo-CWG1 > homo-CWG10 > homo-CpG1 > homo-CpG10 (**Fig 4**). Collectively, our findings show that PARP1 binding is inversely correlated to the methylation status.

### Functional context of PARP1-mediated differentially methylated sites in the genome

Our data suggests that PARP1 is involved in eukaryotic DNA methylation in gene expression, which is consistent with previous studies, concluding that PARP1 through PARylation of DNMT1 determines the methylation pattern at specific promoters [71,75]. We next asked whether PARP1 binding and DNA methylation pattern regulate each other globally. We treated cells with either 5-aza-cytidine or decitabine (both DNA demethylating agents), and measured the occupancy of PARP1 at specific promoters in MCF7 cells. We show that treatment resulted in an increase of PARP1 occupancy at certain promoters (**S5A Fig**), implying that there is interplay between PARP1 and methylation. We then looked at what happens to the methylation status when PARylation is inhibited. Cells were treated with PARylation inhibitor PJ34 and total methylation in cells was measured. Interestingly, we observed no significant change in total methylation content between non-treated and PJ34-treated cells (**S5B and S5C Fig**). We therefore hypothesize that changes in methylation pattern must be genomic region specific.

**Fig 5:**
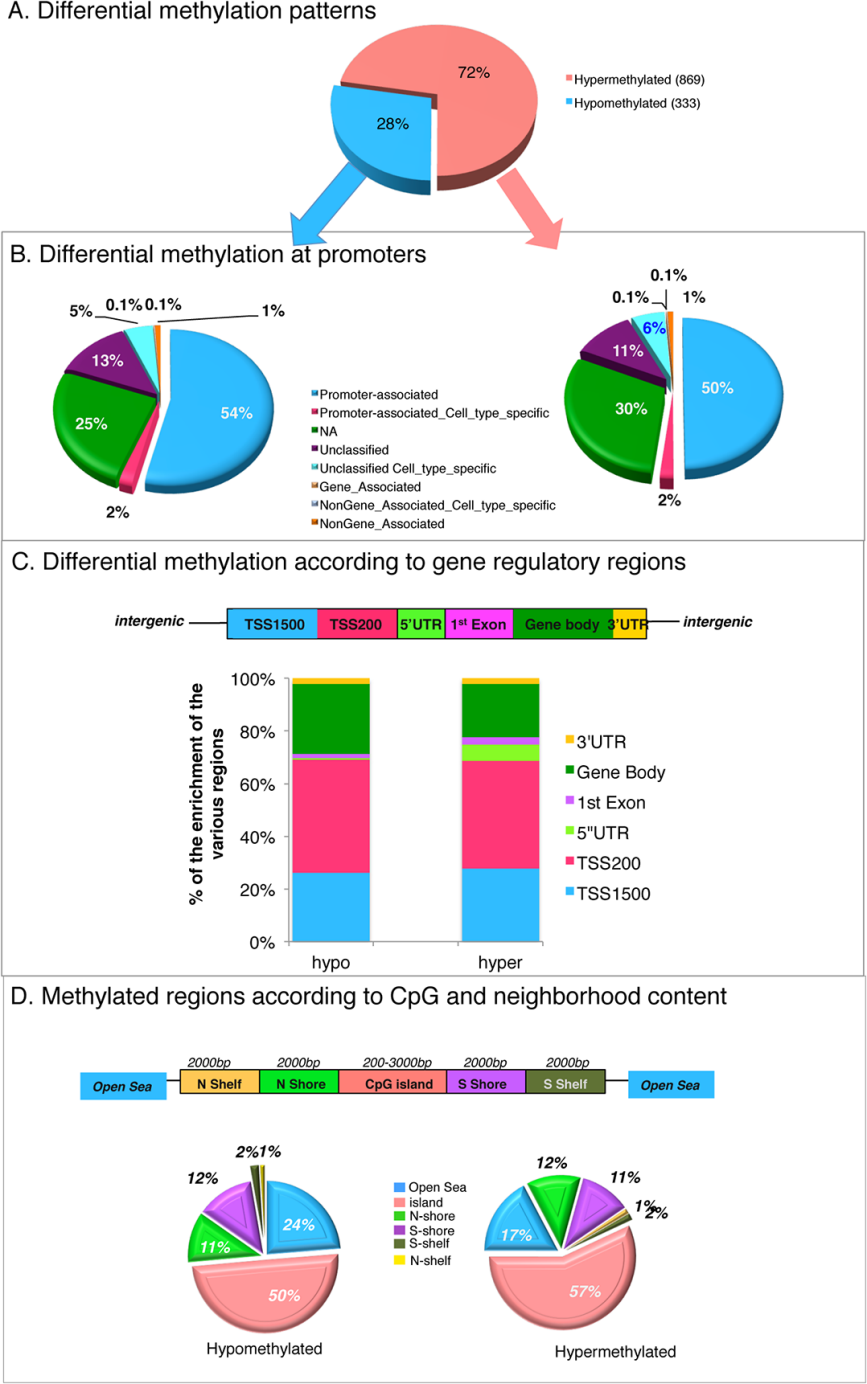
PARP1-mediated differential methylation profile (after PARylation inhibition) according to: (A) all differential methylated patterns as analyzed on the Infinium HumanMethylation450 BeadChip; (B) differential methylated patterns are mapped to gene regions based on their functional genome distribution; (C) CpG island regions based on CpG content and neighborhood context (TSS: proximal promoter, defined as 200 bp or 1500 bp upstream of the transcription start site; UTR: untranslated region; CpG island: 200+ bp stretch of DNA with a C+G content of >50% and an observed CpG/expected CpG in excess of 0.6; shore: the flanking region of CpG islands, 0–2000 bp; shelf: regions flanking island shores (i.e., covering 2000–4000 bp distant from the CpG island); on the HumanMethylation450 BeadChip (two biological replicates of non-treated and PARylation inhibited samples were used).

To determine the PARP1-mediated methylation regions, MCF7 cells were treated with PJ34, the genomic DNA purified and subjected to the Infinium HumanMethylation450 BeadChip microarray analyses. This microarray method allows the interrogation of 485,000 methylation sites per sample at single CpG site resolution [77,78]. After analyses, we detected differential methylation in 1,201 CpG sites, of which 869 were hypermethylated and 333 were hypomethylated (**Fig 5A; S6 Fig, S2 and S3 Tables)**. These differentially methylated regions were non-randomly distributed **(Fig 5A and 5D)** in relationship to gene annotations and CpG density.

From the functional genome distribution standpoint (i.e. distribution promoter, gene body, 3’UTR and intergenic regions), we found that 641 (53.37%) differentially methylated sites were located in promoters, while 243 sites (20.23%) were located in the gene body (**Fig 5B and S2 and S3 Tables**). In addition, 19 (1.58%) and 150 (12.48%) of these differentially methylated sites were found at 3' untranslated regions and intergenic sequences, respectively (**Fig 5B and S7 Fig**). From the CpG content and neighborhood context standpoint, we referred to: (i) “islands” as a DNA sequence (>200 bp window) with a GC content >50% and an observed CpG ratio of >0.6; (ii) “shores” as sequences 0–2 kb distant from the CpG island; (iii) “shelfs” as sequences 2–4 kb distant from the CpG island; and (iv) “open sea/others” as the remaining sequences (**Fig 5C**). Using these criteria, we determined that 661 or 55.04% of the differentially methylated sites were in CpG islands, 280 (23.31%) were in CpG shores, 32 (2.66%) were in CpG shelves and 229 (19.07%) were in the open sea (**Fig 5C**). Globally though, putting the shores and the CpG islands together, PARP1-mediated differential methylation occurred preferentially at these sites. These results highlight PARP1’s critical role in methylation at epigenetically variable regions. Furthermore, most of these sites are at promoters, indicating a role for PARP1 in regulating active transcription (**Fig 5B**). We did not observe any preference in the chromosome with respect to the differential methylation patterns as the differential methylated sites were distributed among all 22 autosomal chromosomes and one sex chromosome (data not shown).

### Gene Ontology analysis of PARP1-mediated hypo and hypermethylated loci

**Fig 6:**
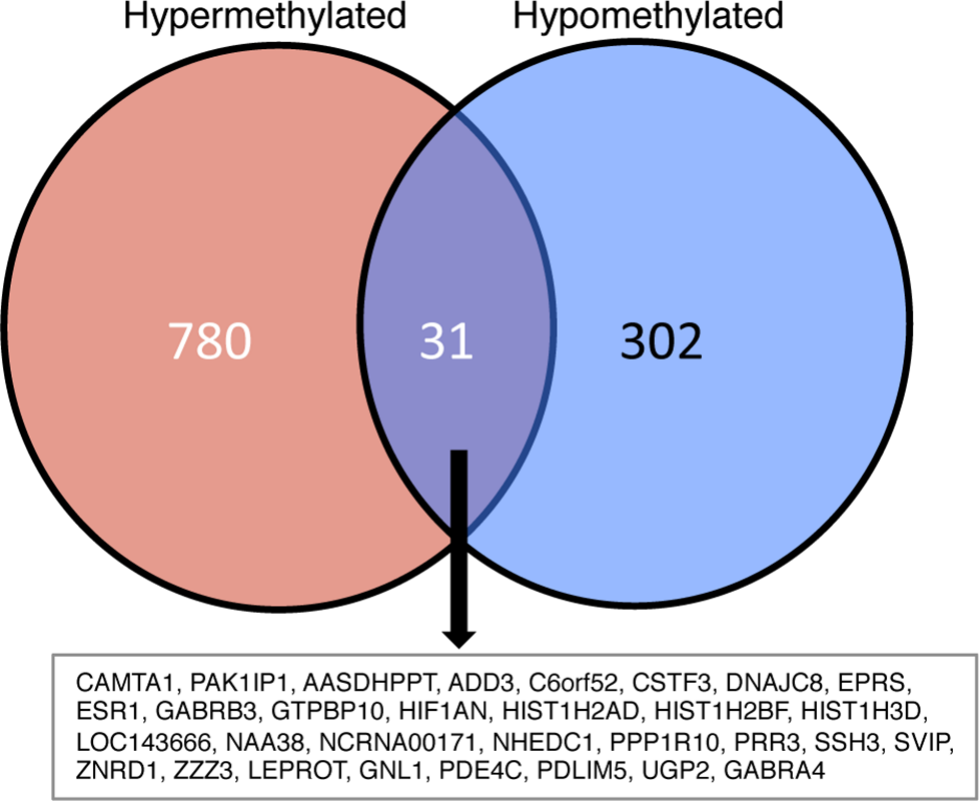
Venn diagram analyses of genes with differential methylation patterns after PARylation inhibition. These analyses show that some genes are commonly modulated by PARylation inhibition. Interestingly, most of them are differentially modified at different genomic regions of the gene. For instance a particular gene is hypermethylated at the promoter region while it is hypomethylated within the gene body. The function of such differential patterns remains to be elucidated.

A total of 869 genes were hypermethylated and 333 genes were hypomethylated in MCF7 cells after treatment with PJ34 **(Fig 6)**. We next asked whether these PARylation-mediated differential methylation patterns were linked to specific biological processes. By performing enrichment analysis using Gene Ontology (GO) and KEGG pathways, we found that target genes of PARylation-mediated methylation are involved in distinct cellular processes (**S4 Table**). In addition, PARylation-mediated hypomethylated genes are involved in significant pathways attributed to cancer related cell events, focal adhesion and spliceosome activities among others. Conversely, hypermethylated genes are involved in pathways involved in adherens junctions, ribosomes, nucleotide excision repair and homologous recombination (**S4 Table**). Interestingly, 39 PARylation-methylation-mediated genes were both hypo- and hypermethylated (**Fig 6**). We hypothesized that these could be differentially methylated at different genomic locations. Further analyses show this to be true (for example: PARylation- mediated hypomethylation of the promoter of PAK1P1 as well as hypermethylation to the gene body) (**S4 Table**). We show for the first time that inhibition of PARylation affects a large number of methylation events at several CpGs of which the promoter regions were most affected.

### Validation of microarray differential methylation analyses

**Fig 7:**
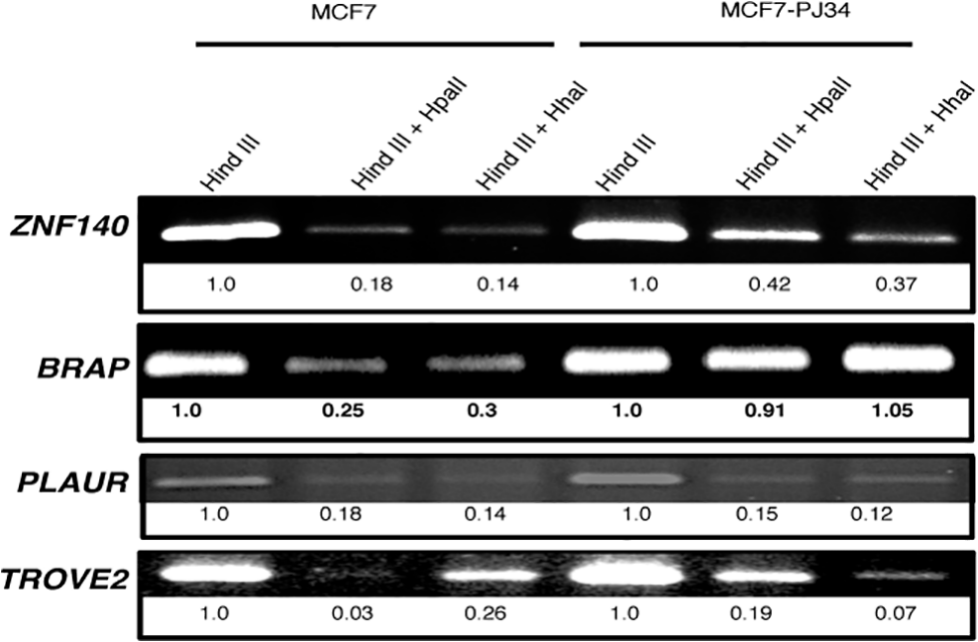
Validation of differential methylated regions mediated by PARylation using MSRE-PCR results on selected genes. Methylation-specific PCR (MSP) assay of three genes in MCF-7 cells (duplicate reactions for each gene). Primers for various genes in NT and PJ34-treated cells at corresponding CpG islands were used. Three biological replicates of samples were used.

We used methylation-sensitive restriction enzyme digestion PCR (MSRE-PCR) [79] with gene-promoter-specific primers to validate the changes in methylation pattern in response to PARylation inhibition. This method fingerprints the methylation patterns by using methylation-sensitive restriction enzymes. First, we normalized the length of genomic DNA fragments, used for MSRE-PCR by digesting DNA with *HindIII* restriction enzyme. Then, these DNA samples were further digested with *HpaII* (CCGG site) and *HhaI* (GCGC site) restriction enzymes, which are inhibited by the methylation of the internal cytosine. Samples were then subjected to PCR amplification, which will only occur when the sites are methylated, and therefore uncut by the two methylation sensitive enzymes. Our analyses show that at the promoter of ZNF140 there is very little methylation in NT cells, but with PJ34 treatment we observed an increase in methylation, as indicated by increase in PCR amplification (**Fig 7**). A similar pattern was observed at the BPAP (BRAC1-associating protein) promoter. Meanwhile at the TROVE2 promoter in NT cells, while the *HpaII* site is unmethylated, the *HhaI* site is methylated. Interestingly, treatment with PJ34 showed a reversal of the methylation pattern at these restriction enzyme sites. All these results are consistent with the microarray analyses. However at the PLAU promoter, MSRE-PCR analyses showed no change in methylation pattern, though our microarray analyses show hypomethylation after PJ34 treatment. We attribute these differences as due to the lower resolution accorded by MSPCR analyses compared to the microarray analyses (**Fig 7**). Finally, since PJ34 is a broad based PARP inhibitor, we verified that the changes in methylation involve PARylation, by using a second PARP inhibitor DPQ. Inhibition of PARylation with DPQ and subsequent MSRE-PCR, showed a similar trend in methylation patterns as with PJ34 (**S8 Fig**).

## Discussion

Though earlier studies have shown PARP1 involvement in chromatin remodeling, transcription activation and repression, a consensus among these functions remains poorly understood. PARP1 spatial localization on the human genome landscape provides extremely valuable insights for understanding how PARP1 coordinates maintenance of chromatin architecture with gene expression regulatory networks. Several studies have demonstrated that nucleosome free regions at TSS aid in promoter accessibility to pre-transcription initiation complexes that are required for transcription initiation [80]. We found PARP1 enriched at TSS of actively transcribed gene promoters in both MCF7 and MDA-MB231 breast cancer cell lines. In contrast, most of the transcriptionally repressed gene promoters were devoid of PARP1. Moreover, Gene Ontology analyses strongly indicate that PARP1 binds at TSSs of genes involved in metabolic processes, developmental processes, cell communication and compartmentalization (data not shown). These results suggest that PARP1 is a global regulator of eukaryotic gene expression.

Gene regulatory regions are characterized by specific histone marks. H3K4me3 and H3K36me3 associate with active promoters and gene bodies respectively [81,82], while H3K27ac differentiate active and poised enhancers [83]. Likewise, H3K9me3 and H3K27me3 have been implicated in repressive chromatin domain formation and gene silencing [84–86]. Our observations of the correlatively binding of PARP1 at regions bound by H3K36me3 and H3K4me3 and its absence at H3K9me3 and H3K27me3 sites is consistent with the idea of the involvement of PARP1 in active chromatin-mediated gene regulatory processes corroborating other studies [87]. While both cell lines exhibited similar association of PARP1 and H3K4me3, the differential association of PARP1 and regions enriched by the other histone marks especially, H3K36me3 and H3K27me3 in these cells might be biologically relevant (**Fig 2A**). We posit that these differences might suggest a functional cell type specific activity that requires further studies to decipher.

DHS and CTCF sites also characterize regulatory regions. Our comparative studies reveal that PARP1 binds to DHSs, as well as regions bound by CTCF both in MCF7 and MB231 cells (**Fig 2B and Fig 3**), further supporting a role of PARP1 in gene regulation. Since PARP1 binds to regions of active chromatin marks, it is not surprising that it associates with DHSs. Interestingly though, the binding of PARP1 to CTCF sites is consistent with previous studies showing that PARP1 forms a complex with CTCF to modulate DNA methylation [71]. Furthermore, it was shown that CTCF-dependent PARP1 automodification and the PARylation of DNMT1 to inhibit its methylation activity may be a possible mechanism for CTCF insulator activity [88,89]. Hence, our correlative studies of genome-wide CTCF and PARP1 binding support the notion that these two factors act together in gene regulation and supports PARP1’s insulator function.

Since earlier studies suggested that PARP1 also PARylates DNMT1 to inhibit DNA methylation, we probed PARP1 binding at CpG sites [73–75]. Our results show that chromatin binding by PARP1 and DNA methylation are mutually exclusive. Conversely highly methylated regions have very stable nucleosomes (**S9 Fig**), which is in concert with previous studies [90,91]. These results may imply that PARP1 or PARylation is involved in a process that stabilizes a non-methylated chromatin region, though a direct test is needed. On the other hand, PARylation has been shown to affect DNMT1 activity [75]. In line with this finding, our data show that after inhibition of PARylation a substantial number of genes were hypermethylated. However, we also observed hypomethylation at other genes. Our data reveal that a balance in methylation globally is important, but also that the interplay between PARP1 and DNA methylation is more complex than just its removal of PARylated DNMT1. Since aberrant changes in DNA methylation have been widely reported in cancer, our data on PARP1’s involvement in DNA methylation provides a platform to further elucidate its effect in gene regulation both in normal and in disease states. A comparison of PARP1-bound nucleosomes to total nucleosomes (MNase-seq) shows that PARP1 binds to a subset of nucleosomes **(S9 Fig).** It is possible that these PARP1 bound chromatin regions are more labile and therefore subject to removal, but this still needs to be tested.

## Conclusion

Overall, our findings suggest that PARP1 binds active gene regulatory regions both in MDA-MB231 and MCF7 cells (**S10 Fig**). PARP1’s absence at methylated DNA and repressive histone marks, and enrichment at active histone modifications and DNase hypersensitive sites indicate that PARP1 communicates with gene expression regulatory networks. Moreover, PARP1 association with CTCF-binding regions indicates involvement in maintenance of chromatin architecture. Finally, the interplay between PARP1’s presence and DNA methylation pattern provides a platform for future studies into the functionality of this interplay and its role in epigenetic regulation in the cell.

## Supporting Information

S1 Fig Reproducibility of PARP-nuc-ChIP-seq data analyses. Using both methods of PARP1 peak calling methods showed high reproducibility with each replicate. (A) Peak detection was performed by running model-based analysis of ChIPSeq (MACS) bandwidth of 200 bp, and a *P*-value threshold of 1 × 10^−5^). (B) Aligning PARP1-nucleosome reads and mapping the centers of these nucleosomes showed reproducibility with each replicate. (C) Wig plots imported into the UCSC browser show reproducibility of the peaks.
(PDF)

S2 Fig ChIP assay and validation. Nuclei from MCF7 and MDA-MB231 cells were formaldehyde-fixed and chromatin MNase digested to yield < 450 bp fragments. Chromatin was subjected to ChIP using PARP1 antibody. (A) Western blot analyses of PARP1 association with the eluted ChIP experiment, validating the presence of PARP1. (B) Resultant DNA fragments elution steps were purified and analyzed on 3% Nusieve^TM^ agarose gel electrophoresis. DNA mononucleosomal fragment (arrow) from elution step was excised and subjected to Illumina sequencing.
(PDF)

S3 Fig PARP1 binding at specific promoters correlate with active gene transcription. We created wiggle files from the PARP1 nuc-ChIP-seq signals and aligned them to the human genome using UCSC genome browser (hg19). PARP1 binding sites in (A) MCF7 and MDA-MB231 in IGFBP7 and (B) IGFBP6 promoters and gene bodies respectively (represented by lines). Measurement of PARP1 occupancy, using quantitative real-time PCR at promoters of (C) IGFBP7 and (D) IGFBP6 respectively. (E) Gene expression profile of IGFBP7 and IGFBP6 as shown in MCF7 and MDA-MB231 obtained from the GSEA (Broad Institute). *Blue bars indicate regions we called based on significance as PARP1-nucleosomes bound.
(PDF)

S4 Fig Evaluation of siRNA transfection efficiency in MCF7 cells. The effects of two PARP1 siRNAs—PARP1-siRNA1 and PARP1-siRNA2, targeting different regions of PARP1 gene on the levels of PARP1 were analyzed by western blot (transfection was performed at 50 nmol/L PARP1 siRNA (Dharmacon) for 48 h). Unrelated siRNA (LacZ) served as negative control. (A) Representative western blot analyses (B) Band intensities were quantified and measured as the percentage over β-actin, normalized to non-treated cells. (C) Representative agarose gel images stained with Gelstar (inverted) of PCR analyses showing the measurement of PARP1 occupancy at PARP1-target genes after PARP1-knockdown. The occupancy of PARP1 at a previously validated promoter (ITPR1 –ref. 29) was used as positive control D) PARP1 occupancy from quantitative real-time PCR analyses at the same PARP1-target genes as in C. Data are expressed as the mean ± S.E.M, n = 3. ** P < 0.001 compared to the control group by student t-test. (PARP1 occupancy in wild-type cells (treated with LacZ) was normalized to 1 and all other measurements of relative PARP1 occupancy in the various treatments were measured relative to PARP1 occupancy in wild-type cells).
(PDF)

S5 Fig Measurement of PARP1 occupancy and activity after 5-aza-cytidine, decitabine and PJ34 treatment. (A) Measurement of PARP1 occupancy at selected promoters after inhibition of methylation using 5-aza-cytidine. (B) Treatment of cells with PJ34 did not change the global percentage of 5-methylcytosine levels in MCF7 and MB-MDA231 cells. (C) Treatment of cells with PJ34, resulted in reduction in PARylation as measured using the Zymo EZ-96DNA Methylation Kit (Catalog #D5004).
(PDF)

S6 Fig Differential methylated pattern analyses analyzed from the Infinium microarray analyses. Scatter plot of log2 (fold_change) of the 1,202 sites illustrating differences in DNA methylation between control and PJ34-treated (PARylation inhibited) cells. A total of **1**,202 CpG Sites with abs(log2(fold_change)) > 1 were observed.
(PDF)

S7 Fig PARylation-mediated methylation patterns in MCF7 cells. Genomic distribution of hypomethylated and hypermethylated according to gene regulatory regions after inhibition of PARylation.
(PDF)

S8 Fig MSRPE PCR on DPQ treated DNA. Results form PARP1 target genes shown in Fig 7 were also validated using a second PARP1-inhibitor (DPQ).
(PDF)

S9 Fig Comparison of MNase-seq data (total nucleosome positioning) with PARP1-bound nucleosomes at (A) TSSs (B) CTCF binding sites (C) highly DNA methylated sites. (PDF)

S10 Fig A view of PARP1 correlative binding with several gene regulatory features. PARP1 chromatin features in MCF7 cells are displayed for a 30 kb region in the AAAS and SP7 locus from UCSC gene annotations. Shown also are total nucleosomes (MNase-seq) from MCF7 cells, used as input. Checkered boxes indicate nucleosomal regions not bound by PARP1. The PARP1-nucleosome- binding sites are shown (1); the gene structure of the genes with this region are shown (2); green bars indicate the CpG islands; 3. Also shown are the DNase hypersensitive sites (4); blue bars show regulatory regions and lastly (6) known CTCF sites.
(PDF)

S1 Table Pearson correlations and *p values* of PARP1 and regulation regions genome-wide and at TSSs. (PDF)

S2 Table Hypermethylated genes and genomic regions mediated by PARylation inhibition. (PDF)

S3 Table Hypomethylated genes and genomic regions mediated by PARylation inhibition. (PDF)

S4 Table Pathways of gene targeted by PARylation-mediated methylation. (PDF)

S5 Table Genes that are both hyper- and hypomethylated by PARylation and the genomic regions affected. (PDF)
