# Supplementary material for: Genome-Wide Profiling of PARP1 Reveals an Interplay with Gene Regulatory Regions and DNA Methylation
Source: PLoS One. 2015 Aug 25;10(8):e0135410. doi: 10.1371/journal.pone.0135410 (PMC4549251; doi:10.1371/journal.pone.0135410)
Supplement: S1 Fig — Using both methods of PARP1 peak calling methods showed high reproducibility with each replicate. (A) Peak detection was performed by running model-based analysis of ChIPSeq (MACS) bandwidth of 200 bp, and a P-value threshold of 1 × 10^−5^). (B) Aligning PARP1-nucleosome reads and mapping the centers of these nucleosomes showed reproducibility with each replicate. (C) Wig plots imported into the UCSC browser show reproducibility of the peaks. (PDF) [file pone.0135410.s001.pdf]

Figure S1

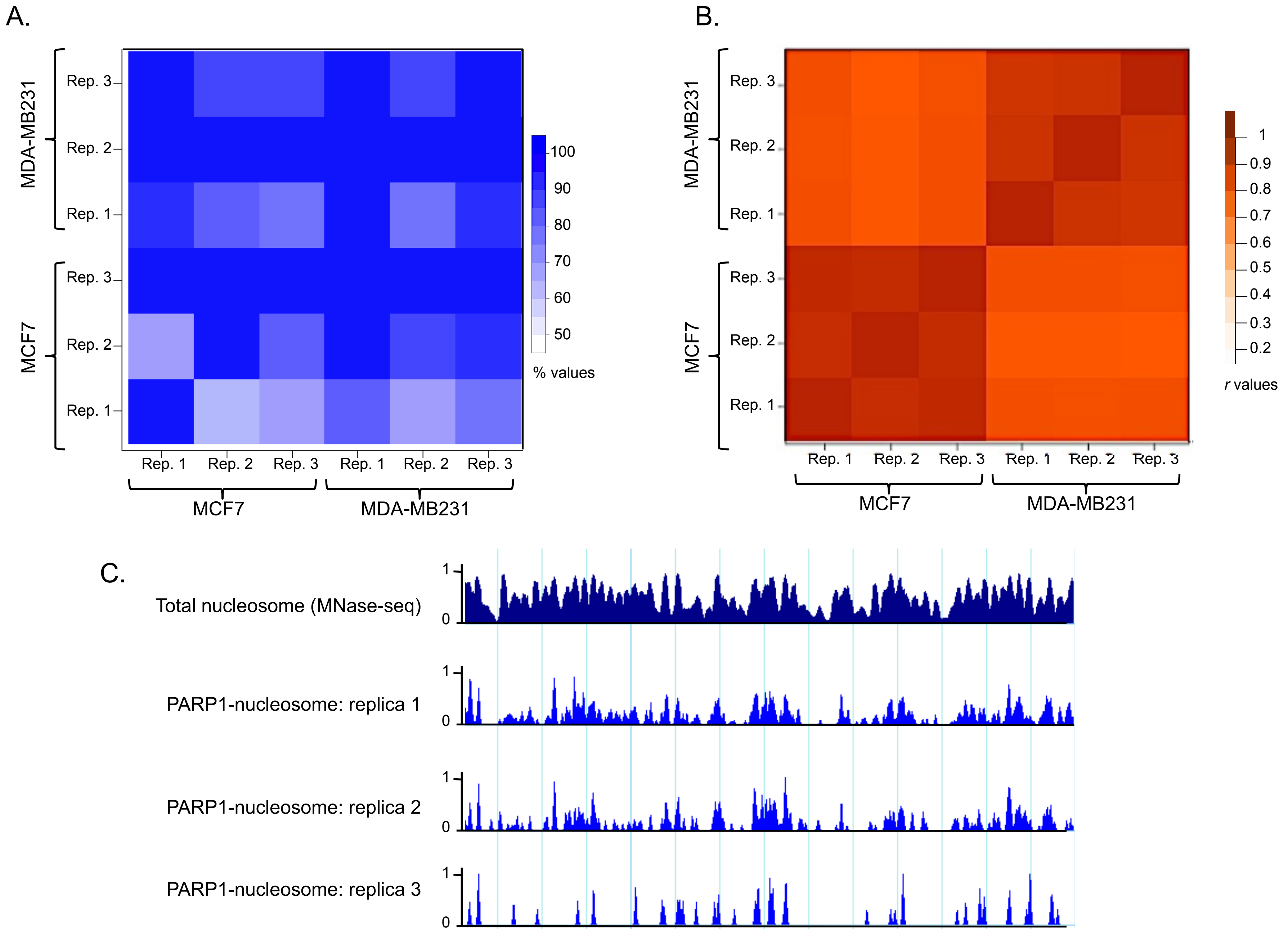

A.
MDA-MB231
MCF7
Rep. 3
Rep. 2
Rep. 1
Rep. 3
Rep. 2
Rep. 1
100
90
80
70
60
50
% values
Rep. 1
Rep. 2
Rep. 3
Rep. 1
Rep. 2
Rep. 3
MCF7
MDA-MB231
B.
MDA-MB231
MCF7
Rep. 3
Rep. 2
Rep. 1
Rep. 3
Rep. 2
Rep. 1
1
0.9
0.8
0.7
0.6
0.5
0.4
0.3
0.2
r values
Rep. 1
Rep. 2
Rep. 3
Rep. 1
Rep. 2
Rep. 3
MCF7
MDA-MB231
C.
Total nucleosome (MNase-seq)
PARP1-nucleosome: replica 1
PARP1-nucleosome: replica 2
PARP1-nucleosome: replica 3
1
0
1
0
1
0
1
0

Figure S1: Reproducibility of PARP1-nuc-ChIP-seq data: Using both methods of PARP1 peak calling methods showed high reproducibility with each replicate. A) Peak detection was performed by running model-based analysis of ChIPSeq (MACS) bandwidth of 200 bp, and a *P*-value threshold of 1×10^{-5}). B) Aligning PARP1-nucleosome reads and mapping the centers of these nucleosomes showed reproducibility with each replicate. C) Wig plots imported into the UCSC browser show reproducibility of the peaks.
