# Supplementary material for: Genome-Wide Profiling of PARP1 Reveals an Interplay with Gene Regulatory Regions and DNA Methylation
Source: PLoS One. 2015 Aug 25;10(8):e0135410. doi: 10.1371/journal.pone.0135410 (PMC4549251; doi:10.1371/journal.pone.0135410)
Supplement: S2 Fig — Nuclei from MCF7 and MDA-MB231 cells were formaldehyde-fixed and chromatin MNase digested to yield < 450 bp fragments. Chromatin was subjected to ChIP using PARP1 antibody. (A) Western blot analyses of PARP1 association with the eluted ChIP experiment, validating the presence of PARP1. (B) Resultant DNA fragments elution steps were purified and analyzed on 3% NusieveTM agarose gel electrophoresis. DNA mononucleosomal fragment (arrow) from elution step was excised and subjected to Illumina sequencing. (PDF) [file pone.0135410.s002.pdf]

Figure S2

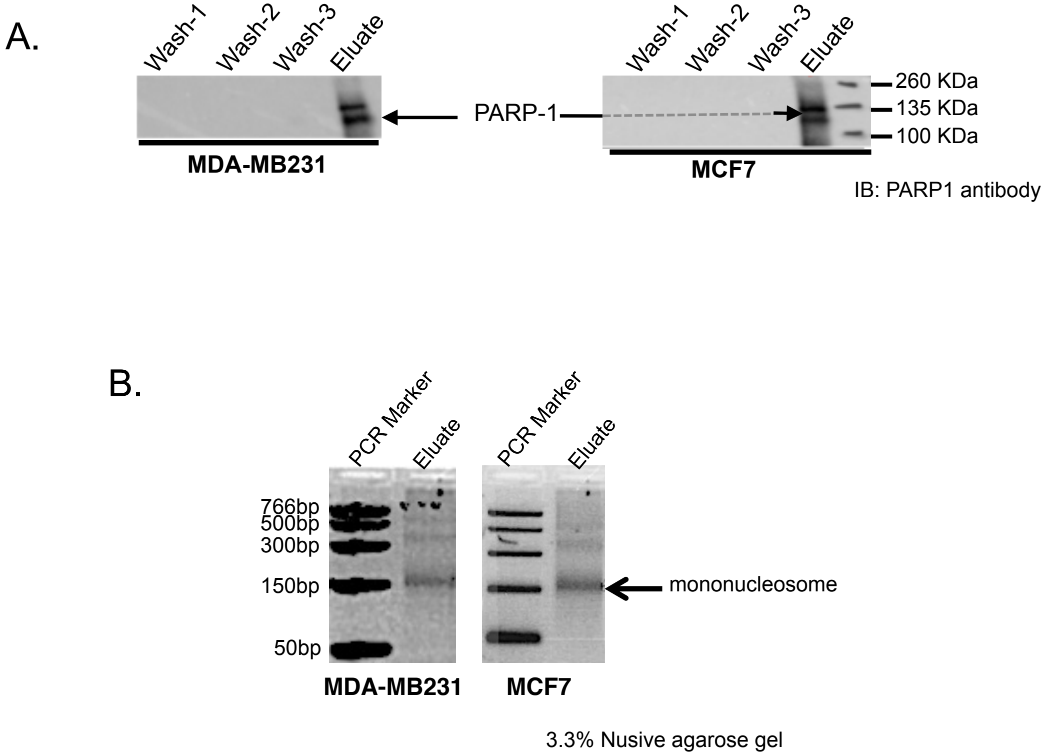

A.
Wash-1
Wash-2
Wash-3
Eluate
PARP-1
260 KDa
135 KDa
100 KDa
MDA-MB231
MCF7
IB: PARP1 antibody
B.
PCR Marker
Eluate
766bp
500bp
300bp
150bp
50bp
mononucleosome
MDA-MB231
MCF7
3.3% Nusive agarose gel

Figure S2: ChIP assay. Nuclei from MCF7 and MDA-MB231 cells were formaldehyde-fixed and chromatin MNase digested to yield < 450 bp fragments. Chromatin was subjected to ChIP using PARP1 antibody. **A)** Western blot analyses of PARP1 association with the eluted ChIP experiment, validating the presence of PARP1. **B)** Resultant DNA fragments elution steps were purified and analyzed on 3% Nusieve^{TM} agarose gel electrophoresis. DNA mononucleosomal fragment (arrow) from elution step was excised and subjected to Illumina sequencing.
