# Supplementary material for: Genome-Wide Profiling of PARP1 Reveals an Interplay with Gene Regulatory Regions and DNA Methylation
Source: PLoS One. 2015 Aug 25;10(8):e0135410. doi: 10.1371/journal.pone.0135410 (PMC4549251; doi:10.1371/journal.pone.0135410)
Supplement: S3 Fig — We created wiggle files from the PARP1 nuc-ChIP-seq signals and aligned them to the human genome using UCSC genome browser (hg19). PARP1 binding sites in (A) MCF7 and MDA-MB231 in IGFBP7 and (B) IGFBP6 promoters and gene bodies respectively (represented by lines). Measurement of PARP1 occupancy, using quantitative real-time PCR at promoters of (C) IGFBP7 and (D) IGFBP6 respectively. (E) Gene expression profile of IGFBP7 and IGFBP6 as shown in MCF7 and MDA-MB231 obtained from the GSEA (Broad Institute). *Blue bars indicate regions we called based on significance as PARP1-nucleosomes bound. (PDF) [file pone.0135410.s003.pdf]

Figure S3

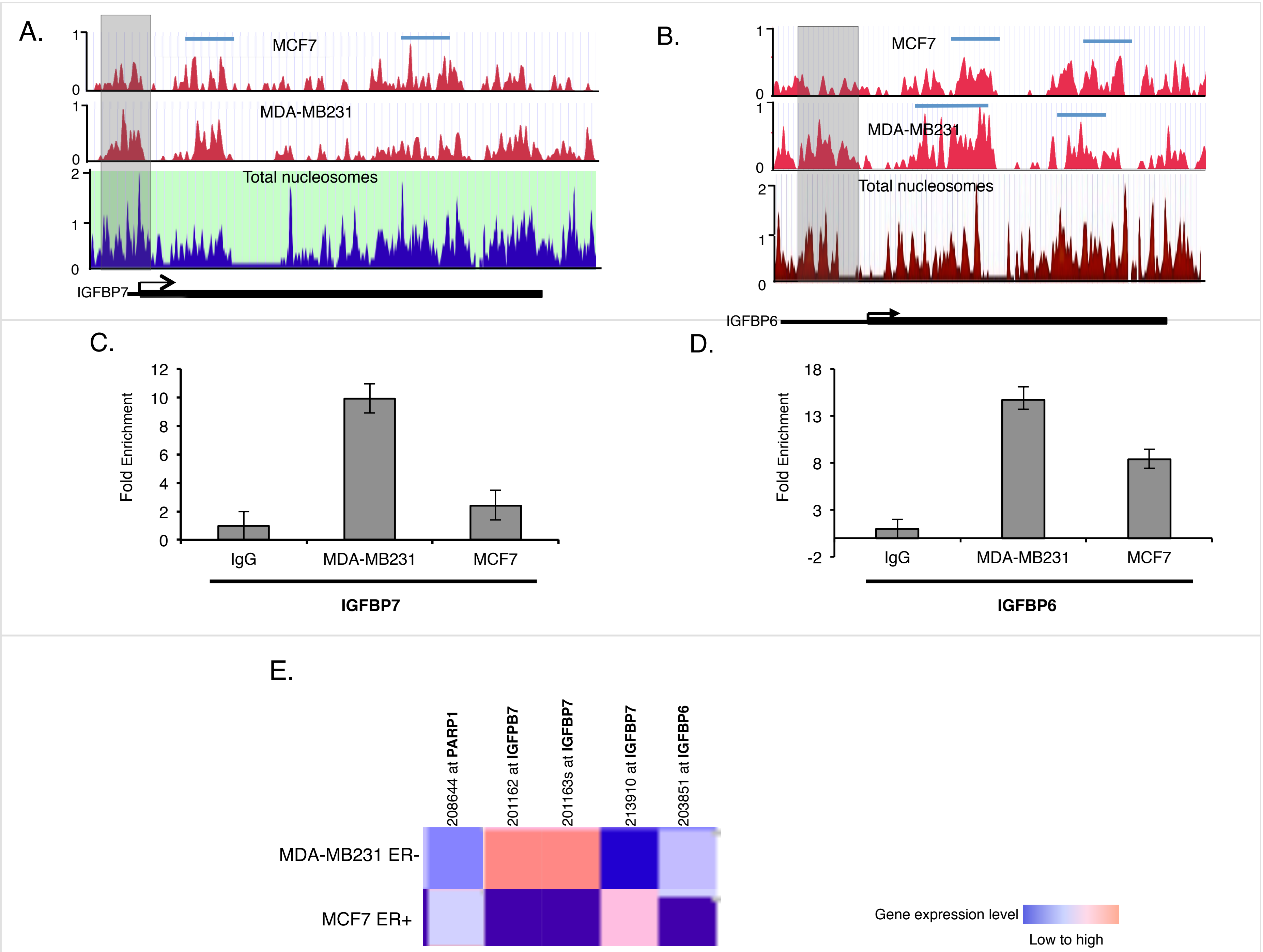

A.
MCF7
MDA-MB231
Total nucleosomes
IGFBP7
B.
MCF7
MDA-MB231
Total nucleosomes
IGFBP6
C.
Fold Enrichment
IgG
MDA-MB231
MCF7
IGFBP7
D.
Fold Enrichment
IgG
MDA-MB231
MCF7
IGFBP6
E.
208644 at PARP1
201162 at IGFPB7
201163s at IGFBP7
213910 at IGFBP7
203851 at IGFBP6
MDA-MB231 ER-
MCF7 ER+
Gene expression level
Low to high

Figure S3: PARP1 binding at specific promoters correlate with active gene transcription. We created wiggle files from the PARP1 nuc-ChIP-seq signals and aligned them to the human genome using UCSC genome browser (hg19). PARP1 binding sites in A) MCF7 and MDA-MB231 in IGFBP7 and B) IGFBP6 promoters and gene bodies respectively (represented by lines). Measurement of PARP1 occupancy using quantitative real-time PCR at promoters of C) IGFBP7 and D) IGFBP6 respectively. E) Gene expression profile of IGFBP7 and IGFBP6 as shown in MCF7 and MDA-MB231 obtained from the GSEA (Broad Institute). * Blue bars indicate regions we called based on significance as PARP1-nucleosomes bound.
