# Supplementary material for: Genome-Wide Profiling of PARP1 Reveals an Interplay with Gene Regulatory Regions and DNA Methylation
Source: PLoS One. 2015 Aug 25;10(8):e0135410. doi: 10.1371/journal.pone.0135410 (PMC4549251; doi:10.1371/journal.pone.0135410)
Supplement: S4 Fig — The effects of two PARP1 siRNAs—PARP1-siRNA1 and PARP1-siRNA2, targeting different regions of PARP1 gene on the levels of PARP1 were analyzed by western blot (transfection was performed at 50 nmol/L PARP1 siRNA (Dharmacon) for 48 h). Unrelated siRNA (LacZ) served as negative control. (A) Representative western blot analyses (B) Band intensities were quantified and measured as the percentage over β-actin, normalized to non-treated cells. (C) Representative agarose gel images stained with Gelstar (inverted) of PCR analyses showing the measurement of PARP1 occupancy at PARP1-target genes after PARP1-knockdown. The occupancy of PARP1 at a previously validated promoter (ITPR1 –ref. 29) was used as positive control D) PARP1 occupancy from quantitative real-time PCR analyses at the same PARP1-target genes as in C. Data are expressed as the mean ± S.E.M, n = 3. ** P < 0.001 compared to the control group by student t-test. (PARP1 occupancy in wild-type cells (treated with LacZ) was normalized to 1 and all other measurements of relative PARP1 occupancy in the various treatments were measured relative to PARP1 occupancy in wild-type cells). (PDF) [file pone.0135410.s004.pdf]

Figure S4

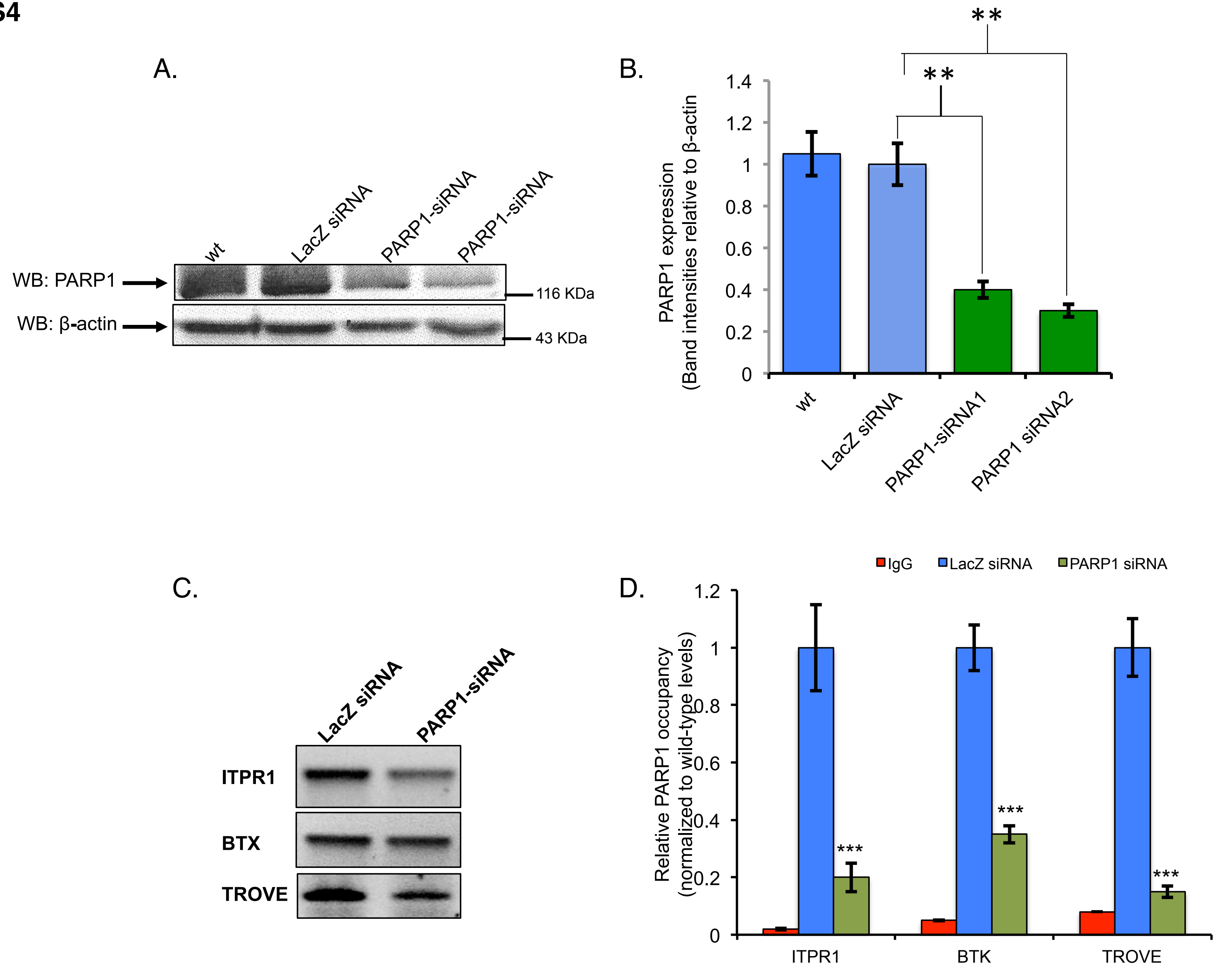
A.
wt
LacZ siRNA
PARP1-siRNA
PARP1-siRNA
WB: PARP1
WB: β-actin
116 KDa
43 KDa
B.
PARP1 expression
(Band intensities relative to β-actin
1.4
1.2
1
0.8
0.6
0.4
0.2
0
wt
LacZ siRNA
PARP1-siRNA1
PARP1 siRNA2
**
**
C.
LacZ siRNA
PARP1-siRNA
ITPR1
BTX
TROVE
D.
IgG
LacZ siRNA
PARP1 siRNA
Relative PARP1 occupancy
(normalized to wild-type levels)
1.2
1
0.8
0.6
0.4
0.2
0
ITPR1
BTK
TROVE
***
***
***

Figure S4: Evaluation of siRNA transfection efficiency in MCF7 cells. The effects of two PARP1 siRNAs - PARP1-siRNA1 and PARP1-siRNA2, targeting different regions of PARP1 gene on the levels of PARP1 were analyzed by western blot (transfection was performed at 50 nmol/L PARP1 siRNA (Dharmacon) for 48 h). Unrelated siRNA (LacZ) served as negative control. **A**. Representative western blot analyses **B**. Band intensities were quantified and measured as the percentage over β−actin, normalized to non-treated cells. **C.** Representative agarose gel images stained with Gelstar® (inverted) of PCR analyses showing the measurement of PARP1 occupancy at PARP1-target genes after PARP1-knockdown. The occupancy of PARP1 at a previously validated promoter (ITPR1 – ref. 29) was used as positive control **D.** PARP1 occupancy from quantitative real-time PCR analyses at the same PARP1-target genes as in C. Data are expressed as the mean ± S.E.M, n = 3. ** $P < 0.001$ compared to the control group by student t-test. (PARP1 occupancy in wild-type cells (treated with LacZ) was normalized to 1 and all other measurements of relative PARP1 occupancy in the various treatments were measured relative to relative PARP1 occupancy in wild-type cells).
