# Supplementary material for: Genome-Wide Profiling of PARP1 Reveals an Interplay with Gene Regulatory Regions and DNA Methylation
Source: PLoS One. 2015 Aug 25;10(8):e0135410. doi: 10.1371/journal.pone.0135410 (PMC4549251; doi:10.1371/journal.pone.0135410)
Supplement: S5 Fig — (A) Measurement of PARP1 occupancy at selected promoters after inhibition of methylation using 5-aza-cytidine. (B) Treatment of cells with PJ34 did not change the global percentage of 5-methylcytosine levels in MCF7 and MB-MDA231 cells. (C) Treatment of cells with PJ34, resulted in reduction in PARylation as measured using the Zymo EZ-96DNA Methylation Kit (Catalog #D5004). (PDF) [file pone.0135410.s005.pdf]

Figure S5

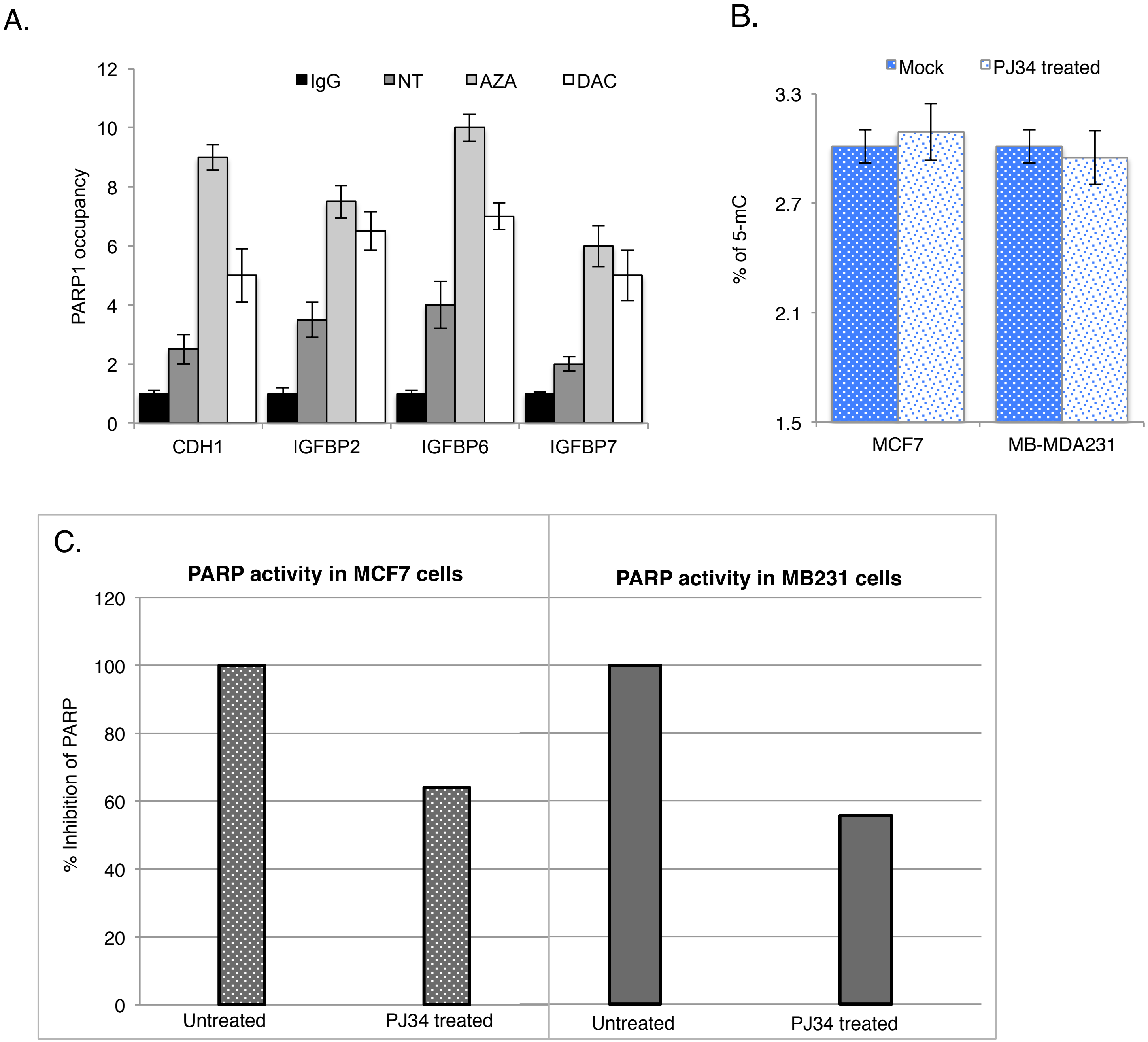

A.
12
10
8
6
4
2
0
PARP1 occupancy
IgG
NT
AZA
DAC
CDH1
IGFBP2
IGFBP6
IGFBP7
B.
Mock
PJ34 treated
3.3
2.7
2.1
1.5
% of 5-mC
MCF7
MB-MDA231
C.
PARP activity in MCF7 cells
PARP activity in MB231 cells
120
100
80
60
40
20
0
% Inhibition of PARP
Untreated
PJ34 treated
Untreated
PJ34 treated

Figure S5: Measurement of PARP1 occupancy and activity after 5-aza-cytidine, decitabine and PJ34 treatment. *A)* Measurement of PARP1 occupancy at selected promoters after inhibition of methylation using 5-aza-cytidine. *B)* Treatment of cells with PJ34, did not change the global percentage of 5-methylcytosine levels in MCF7 and MB-MDA231 cells. *C)* Treatment of cells with PJ34, resulted in reduction in PARylation as measured using the Zymo EZ-96DNA Methylation Kit (Catalog #D5004).
