# Supplementary material for: Genome-Wide Profiling of PARP1 Reveals an Interplay with Gene Regulatory Regions and DNA Methylation
Source: PLoS One. 2015 Aug 25;10(8):e0135410. doi: 10.1371/journal.pone.0135410 (PMC4549251; doi:10.1371/journal.pone.0135410)
Supplement: S6 Fig — Scatter plot of log2 (fold_change) of the 1,202 sites illustrating differences in DNA methylation between control and PJ34-treated (PARylation inhibited) cells. A total of 1,202 CpG Sites with abs(log2(fold_change)) > 1 were observed. (PDF) [file pone.0135410.s006.pdf]

Figure S6

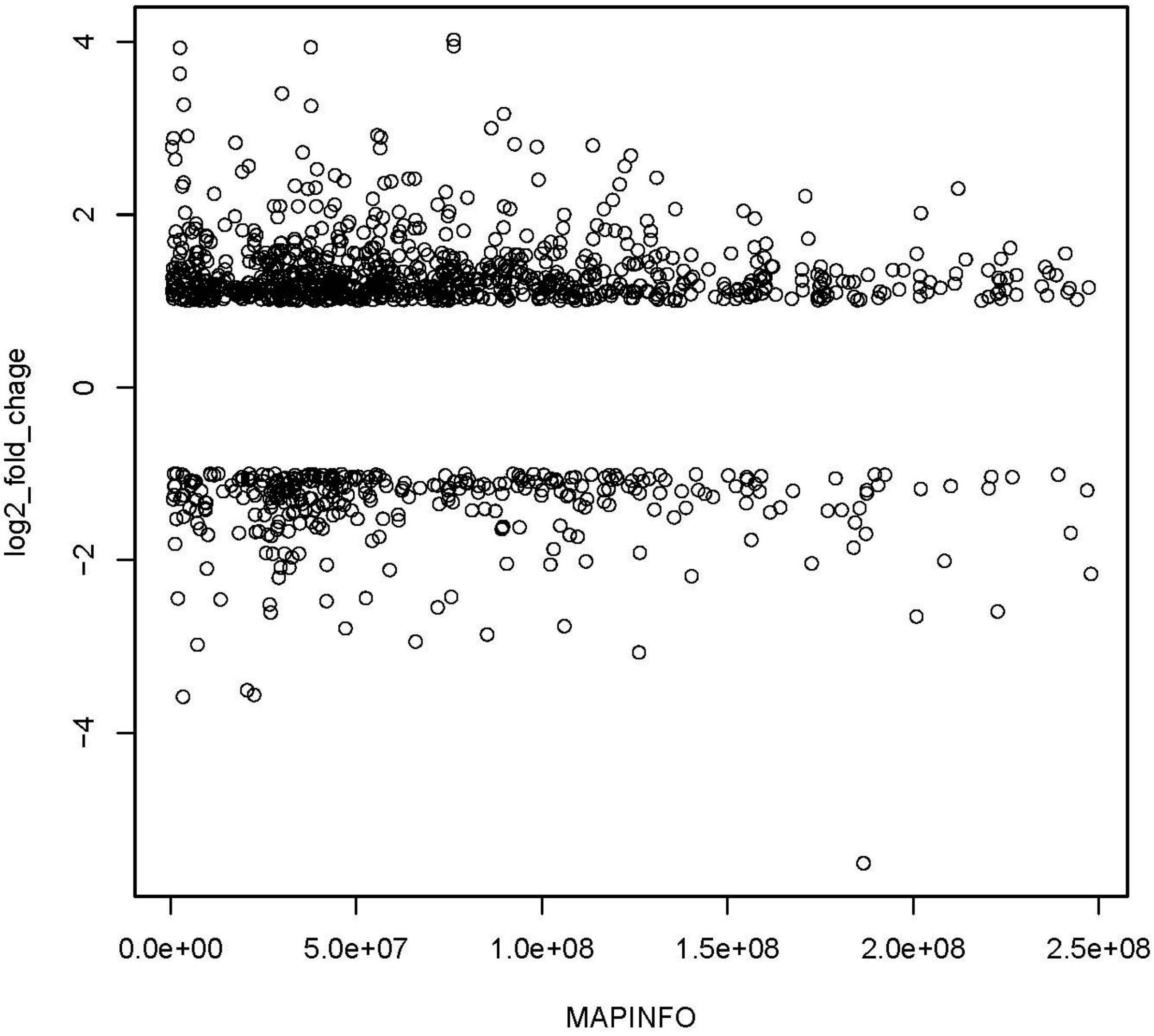

log2_fold_chage
4
2
0
-2
-4
0.0e+00
5.0e+07
1.0e+08
1.5e+08
2.0e+08
2.5e+08
MAPINFO

Figure S6: Differential methylated pattern analyses analyzed from the Infinium microarray analyses. Scatter plot of log2 (fold_change) of the 1,202 sites illustrating differences in DNA methylation between control and PJ34-treated (PARylation inhibited) cells. A total of **1**,202 CpG Sites with abs(log2(fold_change)) > 1 were observed.
