# Supplementary material for: Genome-Wide Profiling of PARP1 Reveals an Interplay with Gene Regulatory Regions and DNA Methylation
Source: PLoS One. 2015 Aug 25;10(8):e0135410. doi: 10.1371/journal.pone.0135410 (PMC4549251; doi:10.1371/journal.pone.0135410)
Supplement: S7 Fig — Genomic distribution of hypomethylated and hypermethylated according to gene regulatory regions after inhibition of PARylation. (PDF) [file pone.0135410.s007.pdf]

Figure S7

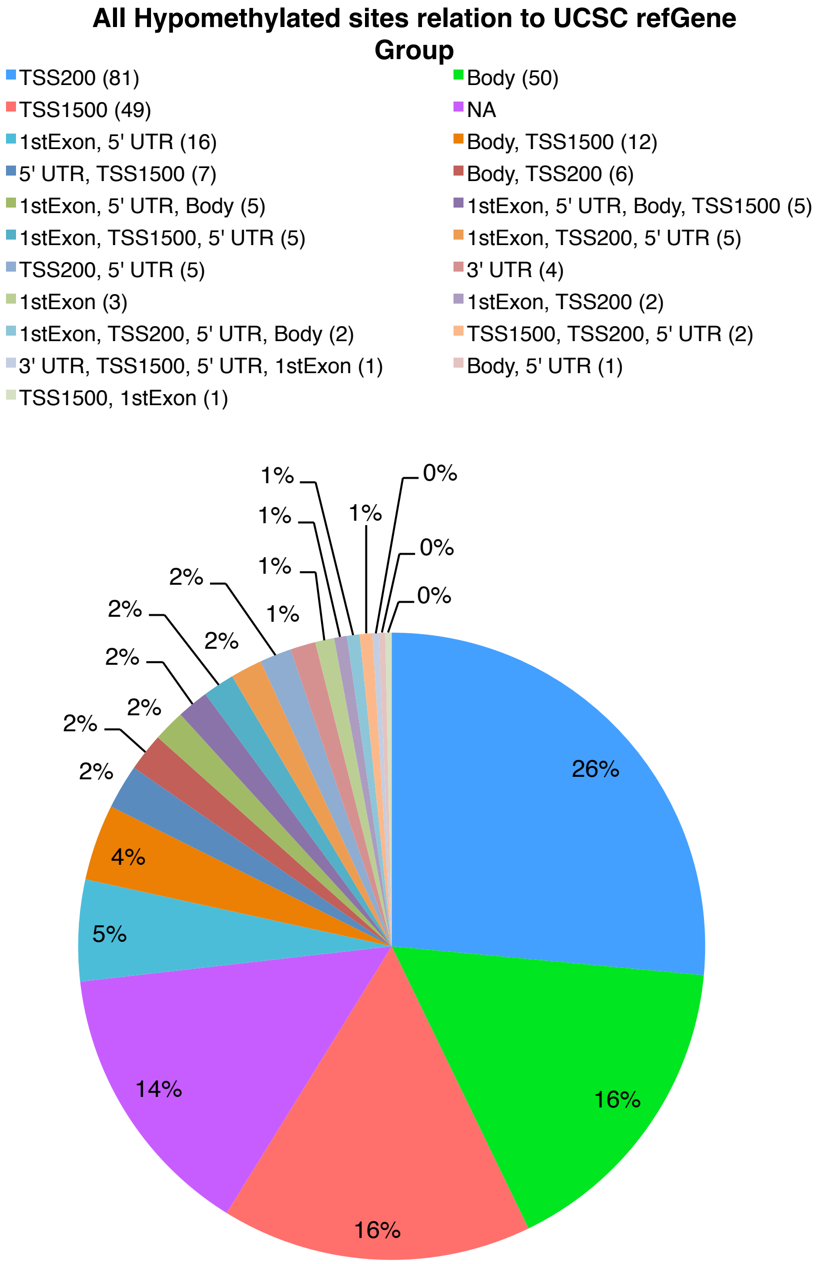
All Hypomethylated sites relation to UCSC refGene Group
TSS200 (81)
Body (50)
TSS1500 (49)
NA
1stExon, 5' UTR (16)
Body, TSS1500 (12)
5' UTR, TSS1500 (7)
Body, TSS200 (6)
1stExon, 5' UTR, Body (5)
1stExon, 5' UTR, Body, TSS1500 (5)
1stExon, TSS1500, 5' UTR (5)
1stExon, TSS200, 5' UTR (5)
TSS200, 5' UTR (5)
3' UTR (4)
1stExon (3)
1stExon, TSS200 (2)
1stExon, TSS200, 5' UTR, Body (2)
TSS1500, TSS200, 5' UTR (2)
3' UTR, TSS1500, 5' UTR, 1stExon (1)
Body, 5' UTR (1)
TSS1500, 1stExon (1)
26%
16%
16%
14%
5%
4%
2%
2%
2%
2%
2%
2%
2%
1%
1%
1%
1%
1%
0%
0%
0%

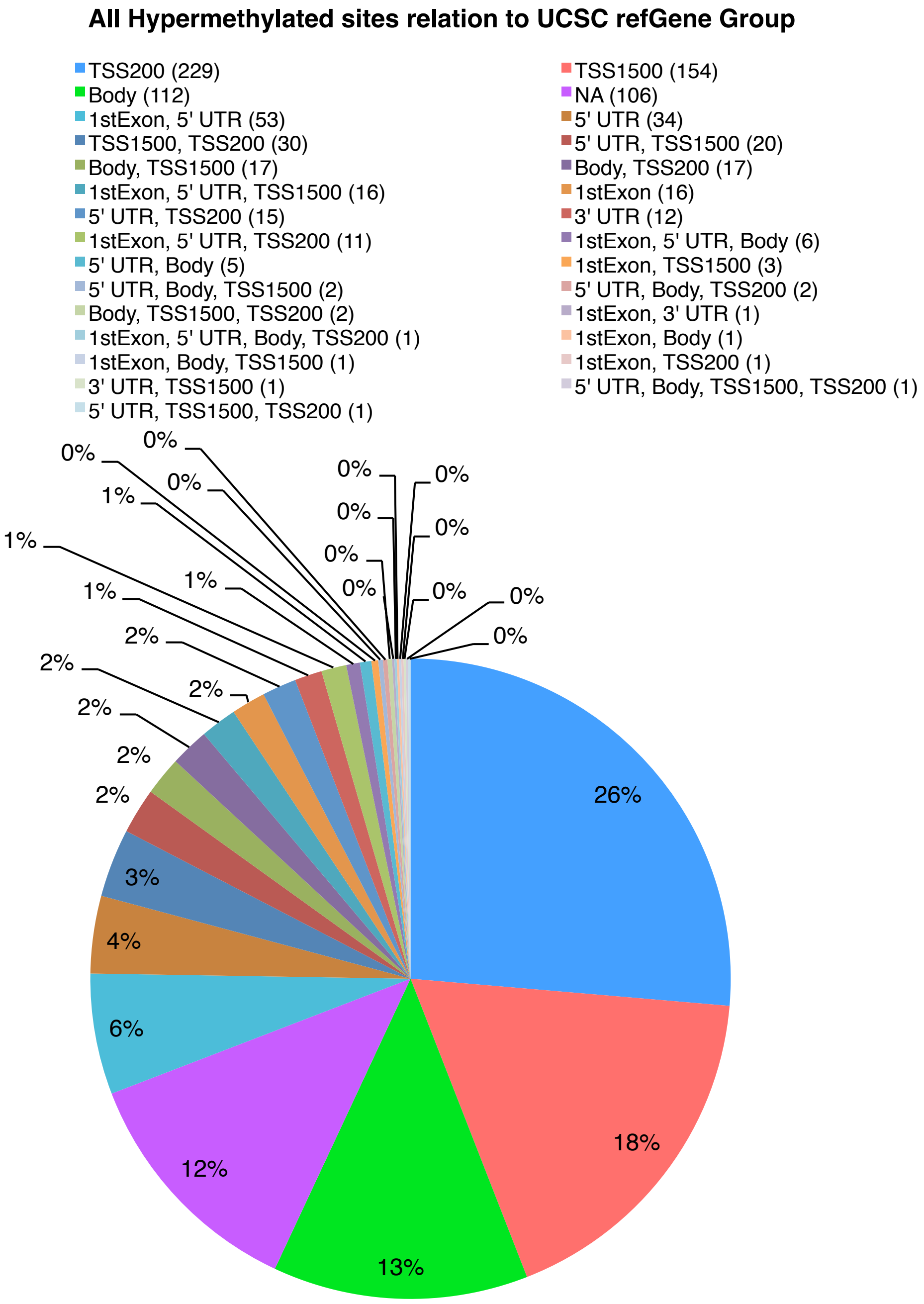
All Hypermethylated sites relation to UCSC refGene Group
TSS200 (229)
TSS1500 (154)
Body (112)
NA (106)
1stExon, 5' UTR (53)
5' UTR (34)
TSS1500, TSS200 (30)
5' UTR, TSS1500 (20)
Body, TSS1500 (17)
Body, TSS200 (17)
1stExon, 5' UTR, TSS1500 (16)
1stExon (16)
5' UTR, TSS200 (15)
3' UTR (12)
1stExon, 5' UTR, TSS200 (11)
1stExon, 5' UTR, Body (6)
5' UTR, Body (5)
1stExon, TSS1500 (3)
5' UTR, Body, TSS1500 (2)
5' UTR, Body, TSS200 (2)
Body, TSS1500, TSS200 (2)
1stExon, 3' UTR (1)
1stExon, 5' UTR, Body, TSS200 (1)
1stExon, Body (1)
1stExon, Body, TSS1500 (1)
1stExon, TSS200 (1)
3' UTR, TSS1500 (1)
5' UTR, Body, TSS1500, TSS200 (1)
5' UTR, TSS1500, TSS200 (1)
26%
18%
13%
12%
6%
4%
3%
2%
2%
2%
2%
2%
2%
1%
1%
1%
1%
0%
0%
0%
0%
0%
0%
0%
0%
0%
0%
0%
0%

Figure S7: PARylation mediated methylation patterns in MCF7 cells. Genomic distribution of hypomethylated and hypermethylated according to gene regulatory regions after inhibition of PARylation.
