# Supplementary material for: Genome-Wide Profiling of PARP1 Reveals an Interplay with Gene Regulatory Regions and DNA Methylation
Source: PLoS One. 2015 Aug 25;10(8):e0135410. doi: 10.1371/journal.pone.0135410 (PMC4549251; doi:10.1371/journal.pone.0135410)
Supplement: S8 Fig — Results form PARP1 target genes shown in Fig 7 were also validated using a second PARP1-inhibitor (DPQ). (PDF) [file pone.0135410.s008.pdf]

Figure S8

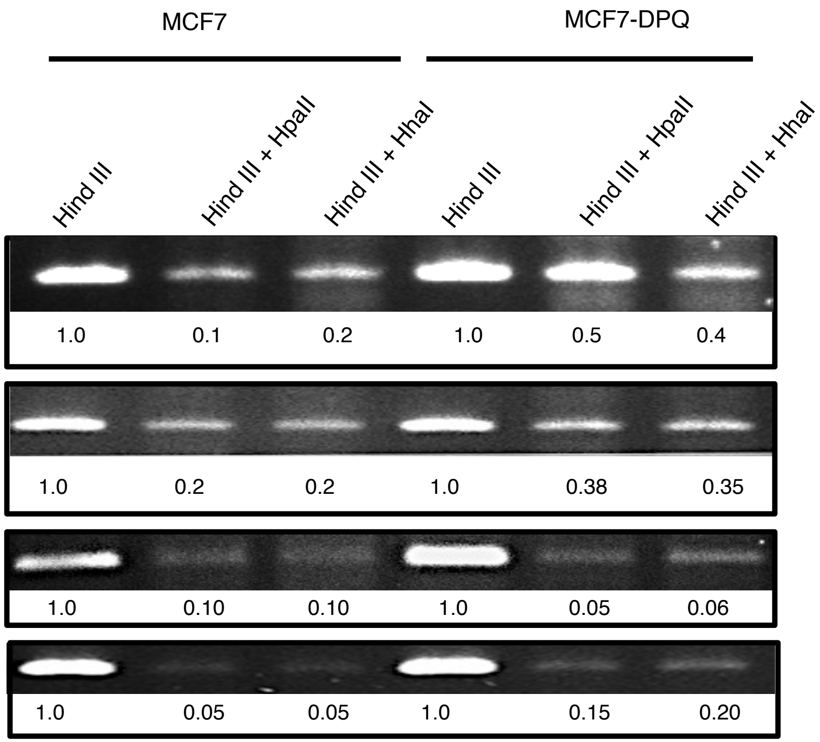
MCF7
MCF7-DPQ
Hind III
Hind III + HpaII
Hind III + HhaI
Hind III
Hind III + HpaII
Hind III + HhaI
1.0 0.1 0.2 1.0 0.5 0.4
1.0 0.2 0.2 1.0 0.38 0.35
1.0 0.10 0.10 1.0 0.05 0.06
1.0 0.05 0.05 1.0 0.15 0.20

Figure S8: MSRPE PCR on DPQ treated DNA. Results form PARP1 target genes shown in Fig. 7 were also validated using a second PARP1-inhibitor (DPQ).
