# Supplementary material for: Genome-Wide Profiling of PARP1 Reveals an Interplay with Gene Regulatory Regions and DNA Methylation
Source: PLoS One. 2015 Aug 25;10(8):e0135410. doi: 10.1371/journal.pone.0135410 (PMC4549251; doi:10.1371/journal.pone.0135410)
Supplement: S9 Fig — (PDF) [file pone.0135410.s009.pdf]

# Figure S9

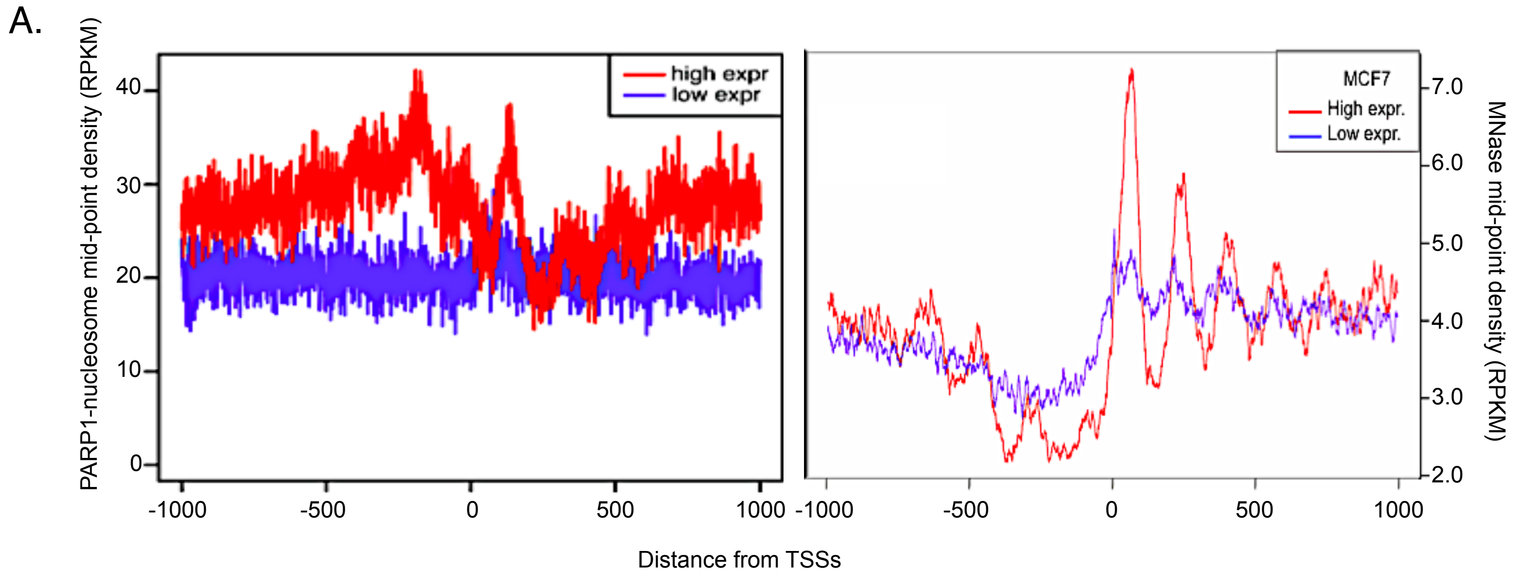
A.
PARP1-nucleosome mid-point density (RPKM)
40
30
20
10
0
high expr
low expr
-1000
-500
0
500
1000
MCF7
High expr.
Low expr.
7.0
6.0
5.0
4.0
3.0
2.0
MNase mid-point density (RPKM)
-1000
-500
0
500
1000
Distance from TSSs

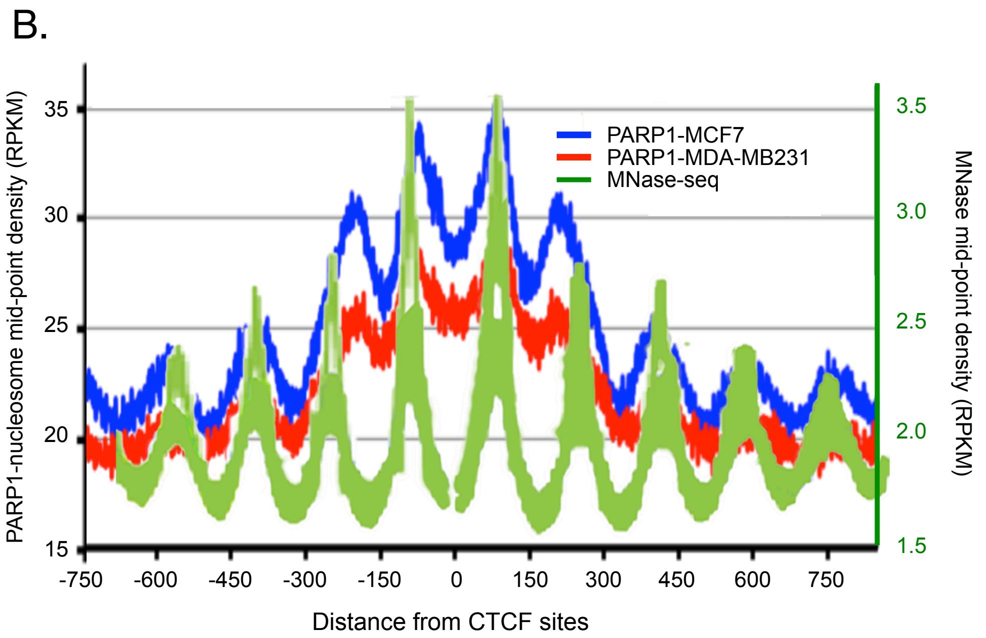
B.
PARP1-nucleosome mid-point density (RPKM)
35
30
25
20
15
PARP1-MCF7
PARP1-MDA-MB231
MNase-seq
3.5
3.0
2.5
2.0
1.5
MNase mid-point density (RPKM)
-750
-600
-450
-300
-150
0
150
300
450
600
750
Distance from CTCF sites

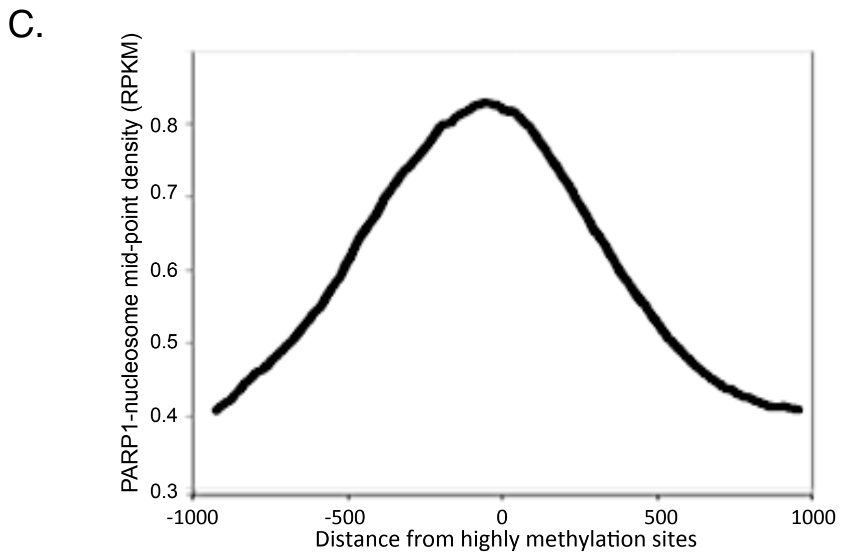
C.
PARP1-nucleosome mid-point density (RPKM)
0.8
0.7
0.6
0.5
0.4
0.3
-1000
-500
0
500
1000
Distance from highly methylation sites

**Figure S9**: Comparison of MNase-seq data (total nucleosome positioning) with PARP1-bound nucleosomes at A) TSSs B) CTCF binding sites C) highly DNA methylated sites.
