# Supplementary material for: Genome-Wide Profiling of PARP1 Reveals an Interplay with Gene Regulatory Regions and DNA Methylation
Source: PLoS One. 2015 Aug 25;10(8):e0135410. doi: 10.1371/journal.pone.0135410 (PMC4549251; doi:10.1371/journal.pone.0135410)
Supplement: S10 Fig — PARP1 chromatin features in MCF7 cells are displayed for a 30 kb region in the AAAS and SP7 locus from UCSC gene annotations. Shown also are total nucleosomes (MNase-seq) from MCF7 cells, used as input. Checkered boxes indicate nucleosomal regions not bound by PARP1. The PARP1-nucleosome- binding sites are shown (1); the gene structure of the genes with this region are shown (2); green bars indicate the CpG islands; 3. Also shown are the DNase hypersensitive sites (4); blue bars show regulatory regions and lastly (6) known CTCF sites. (PDF) [file pone.0135410.s010.pdf]

Figure S10

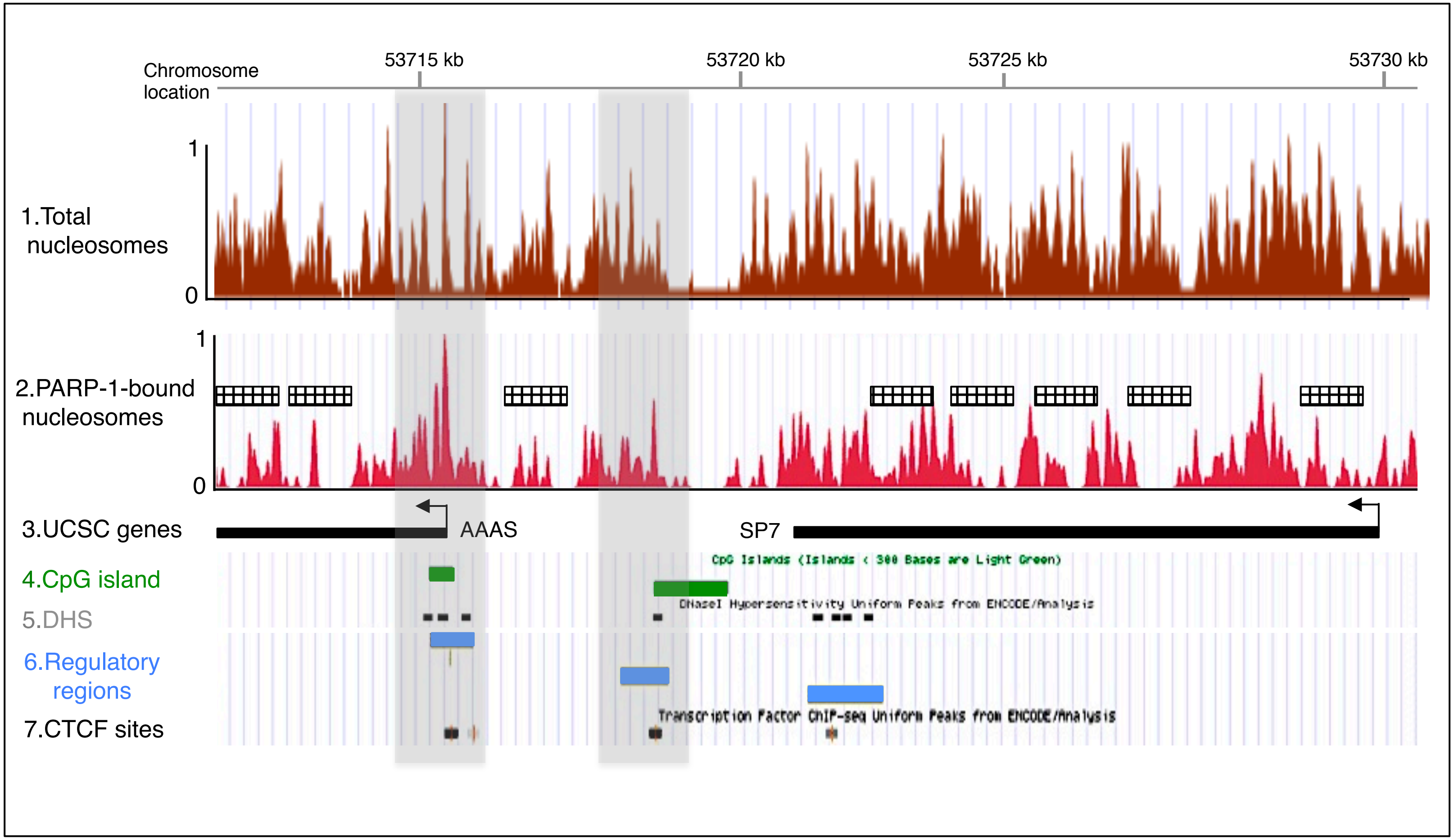
Chromosome location
53715 kb
53720 kb
53725 kb
53730 kb
1
0
1.Total nucleosomes
1
0
2.PARP-1-bound nucleosomes
3.UCSC genes
AAAS
SP7
4.CpG island
5.DHS
6.Regulatory regions
7.CTCF sites
CpG Islands (Islands < 300 Bases are Light Green)
DNaseI Hypersensitivity Uniform Peaks from ENCODE/Analysis
Transcription Factor ChIP-seq Uniform Peaks from ENCODE/Analysis

Figure S10: A view of PARP1 correlative binding with several gene regulatory features. PARP1 chromatin features in MCF7 cells, are displayed for a 30 kb region in the AAAS and SP7 locus from UCSC gene annotations. Shown also are total nucleosomes (MNase-seq) from MCF7 cells, used as input. Checkered boxes indicate nucleosomal regions not bound by PARP1. The PARP1-nucleosome-binding sites are shown (1); the gene structure of the genes with this region are shown (2); green bars indicate the CpG islands; 3. Also shown are the DNase hypersensitive sites (4); blue bars show regulatory regions and lastly (6) known CTCF sites.
