# Supplementary material for: Genome-Wide Profiling of PARP1 Reveals an Interplay with Gene Regulatory Regions and DNA Methylation
Source: PLoS One. 2015 Aug 25;10(8):e0135410. doi: 10.1371/journal.pone.0135410 (PMC4549251; doi:10.1371/journal.pone.0135410)
Supplement: S1 Table — (PDF) [file pone.0135410.s011.pdf]

Table S1: PARP1 correlations with different epigenetic regulators at transcription start site as well as genome-wide. For genome wide, the genome was tiled into 2 KB, and identical calculations as for the TSSs were done.

	**corr_value**	**p_value**
**1. CTCF**<br>**i) PARP1 vs CTCF at TSS**		
ctcf_r1_v_parp1_mcf7	0.11382852	5.126472e-12
ctcf_r2_v_parp1_mcf7	0.19594551	2.2392e-12
ctcf_r1_v_parp1_mdamb231	0.04467774	7.327472e-9
ctcf_r2_v_parp1_mdamb231	0.08303212	1.1311e-10
**ii) PARP1 vs CTCF genome-wide**		
ctcf_r1_v_parp1_mcf7	0.1854415	1.3333e-15
ctcf_r2_v_parp1_mcf7	0.2390277	2.1122e-15
ctcf_r1_v_parp1_mdamb231	0.1608382	1.93178e-13
ctcf_r2_v_parp1_mdamb231	0.1793969	1.327472e-12
**2.Histone modifications**<br>**i) PARP1 vs Histone Marks at TSS**		
H3k9me3_v_parp1_mcf7	0.2667278	1.444064e-15
H3k27ac_v_parp1_mcf7	0.3056719	1.018670e-09
H3k27me3_v_parp1_mcf7	0.3466788	2.174444e-13
H3k36me3_v_parp1_mcf7	0.3065474	1.451234e-08
H3k4me3_r1_v_parp1_mcf7	0.3869864	1.651546e-09
H3k4me3_r2_v_parp1_mcf7	0.3607083	2.211134e-12
H3k9me3_v_parp1_mdamb231	0.1674508	1.939051e-08
H3k27ac_v_parp1_mdamb231	0.1899501	7.041781e-18
H3k27me3_v_parp1_mdamb231	0.1133071	2.378452e-10
H3k36me3_v_parp1_mdamb231	0.2365975	5.312111e-13
H3k4me3_r1_v_parp1_mdamb231	0.3623120	4.614523e-18
H3k4me3_r2_v_parp1_mdamb231	0.3497517	8.111123e-15
**ii) PARP1 vs Histone Marks genome-wide**		
H3k9me3_v_parp1_mcf7	0.4098493	2.1038e-10
H3k27ac_v_parp1_mcf7	0.4011359	1.35210e-12
H3k27me3_v_parp1_mcf7	0.4200107	1.562347e-15
H3k36me3_v_parp1_mcf7	0.3882289	5.11112e-15
H3k4me3_r1_v_parp1_mcf7	0.1566817	7.123415e-08
H3k4me3_r2_v_parp1_mcf7	0.1180702	2.32211e-11
H3k9me3_v_parp1_mdamb231	0.2499175	1.158082e-149
H3k27ac_v_parp1_mdamb231	0.2573392	2.005263e-09
H3k27me3_v_parp1_mdamb231	0.1890674	2.758508e-174
H3k36me3_v_parp1_mdamb231	0.2859072	3.111123e-10
H3k4me3_r1_v_parp1_mdamb231	0.2524751	8.423001e-13
H3k4me3_r2_v_parp1_mdamb231	0.2344856	1.203512e-12
**3. Methylation**<br>**i) PARP1 vs methylation status at TSS**		
methyl_r1_v_parp1_mcf7	0.01473731	1.081047e-17
methyl_r2_v_parp1_mcf7	0.01549252	2.23332e-20
methyl_r1_v_parp1_mdamb231	-0.102433957	1.00549e-16
methyl_r2_v_parp1_mdamb231	-0.092442113	1.00009e-24
**ii) PARP1 vs methylation status genome-wide**		
methyl_r1_v_parp1_mcf7	0.048287217	4.125970e-13
methyl_r2_v_parp1_mcf7	0.028169880	2.067856e-10
methyl_r1_v_parp1_mdamb231	0.017934524	1.149546e-09
methyl_r2_v_parp1_mdamb231	0.001891696	5.423150e-01
