# Supplementary material for: Genome-Wide Profiling of PARP1 Reveals an Interplay with Gene Regulatory Regions and DNA Methylation
Source: PLoS One. 2015 Aug 25;10(8):e0135410. doi: 10.1371/journal.pone.0135410 (PMC4549251; doi:10.1371/journal.pone.0135410)
Supplement: S2 Table — (PDF) [file pone.0135410.s012.pdf]

Table S2: HYPERMETHYLATED GENES AND GENOMIC REGIONS MEDIATED BY PARYLATION INHIBITION

GENE BODY					
ABCA7	AES	AFF1	API5	ARPC3	ASGR1
ATRN	B3GAT3	BAZ2A	BBS4	BTBD8	C13orf38
C20orf114	C20orf20	C20orf95	C21orf66	C4orf32	C7orf28A
CACNA2D1	CACNB1	CAMTA1	CASP12	CCDC138	CCDC24
CERKL	CNPY3	CYP39A1	DACT1	DEAF1	DUSP12
EDIL3	EFCAB5	ERGIC3	ERICH1	EVI5	EXPH5
FAM45B	FDFT1	FLJ35220	GAA	GABBR1	GAS7
GFOD1	GLYR1	GRIA4	GRID2IP	GRK5	GUCY2G
H2AFZ	HCN1	HSPA12A	HSPA8	KCNJ9	KHDRBS2
KIF13B	KLF13	KLHL1	KPNA6	LOC100133612	LOC100134368
LOC100302652	LOC344967	LOC399815	LOC440839	LOC642846	LOC642846
LOC729082	LONRF2	MAN2A1	MCF2L	MDN1	MKX
MMP17	MPL	MTA3	MYO10	NAA16	NCRNA00085
NTN1	OPCML	OR7E5P	OR8U8	ORC6L	PAK1IP1
PAPSS1	PDCD5	PDXDC2	PICALM	PINK1	PITPNB
PLAU	PLAUR	PLK1	PRX	PTPRD	RABGAP1L
RNF166	RNPEP	ROR1	RPLP1	RPS24	SARNP
SCFD2	SGCD	SLC25A38	SNHG1	SORL1	SPNS2
TCF25	TEPP	THBS4	TLE2	TMCO6	TMEM167A
TMEM87B	TRIM31	TRIP10	UBR3	UNC5D	USP22
VGLL4	WDFY2	WDR60	ZFAND1	ZFYVE28	ZNF214
ZNF511					

PROMOTER					
AASDHPPT	ABHD4	ABLIM2	ACAD10	ACADSB	ACAP3
ACAT2	ACBD5	ACIN1	ACTG1	ACTR6	ADAM32
ADD3	ADRBK1	AFG3L1	AGPHD1	AHI1	AK2
AK5	AKAP13	AKR1A1	AKTIP	ALG9	ALMS1
AMPH	ANG	ANKMY1	ANKRD34B	ANP32A	ANXA3
AP1S3	AP2M1	APAF1	APBA3	APBB3	APOL2
ARHGAP28	ARL1	ARL5A	ARSA	ARSK	ASB3
ASNSD1	ASPA	ATAD1	ATHL1	ATN1	ATP10D
ATP5I	ATP6V0A1	ATP6V1G2	AURKB	BAT4	BDH1
BMS1	BNIP2	BPNT1	BRAP	BRD2	BTBD18
BTF3L4	BTRC	C10orf55	C12orf11	C12orf32	C12orf65
C14orf119	C15orf40	C15orf42	C15orf63	C17orf100	C18orf19
C18orf54	C19orf62	C1orf151	C1orf174	C1orf182	C1orf212
C1orf31	C1orf43	C1orf85	C20orf111	C20orf20	C21orf45
C21orf49	C22orf32	C2orf47	C2orf67	C4orf46	C6orf114
C6orf126	C6orf52	C8orf40	C8orf59	CABYR	CALB2
CALU	CAMTA2	CANX	CAPS2	CBFB	CBLB
CBLN2	CBR4	CBR4	CBY1	CCAR1	CCDC159
CCDC92	CCT3	CD320	CDC20	CDC27	CDC42
CDC7	CDK12	CDKN1C	CENPBD1	CEP290	CHAC2
CHEK1	CHORDC1	CIZ1	CLIC5	CLPB	CMTM4
CNNM2	CNP	COG3	COPB1	CPSF6	CSNK2B
CSTF3	CTDSPL2	CTU2	CWF19L1	CYB5RL	CYBASC3
DBP	DBR1	DCAF10	DCBLD2	DCUN1D2	DDA1
DDB1	DDX17	DDX24	DEK	DEM1	DFFA

DFFB	DHCR7	DHDDS	DHRS12	DHX15	DHX37
DMC1	DNAH14	DNAH3	DNAH6	DNAJC21	DNAJC3
DNAJC5B	DNAJC8	DPEP2	DPYSL4	DSE	DUT
EED	EEF1A1	EEF1E1	EFEMP2	EFHC1	EIF4A1
ELP2P	ENKUR	EPHX1	EPRS	ERCC2	ERCC5
ERGIC2	ERO1LB	ESR1	ETFDH	ETS1	ETV3
ETV4	EXO1	EXOSC9	FAM126A	FAM149B1	FAM160B1
FAM24B	FAM43A	FAM46A	FAM49B	FANCI	FARSB
FBXO2	FBXO4	FBXO44	FBXO47	FBXO8	FCER1A
FDXR	FGFR1OP2	FLJ41603	FLJ44606	FMNL2	FOXM1
FOXP1	FRS3	FUZ	GABRB3	GALNT7	GARS
GATA2	GEMIN4	GFER	GJD3	GLMN	GLRA3
GMIP	GNB4	GNG12	GNG5	GNG7	GNPNAT1
GORASP1	GPR108	GPR176	GPR19	GPR4	GPR63
GTPBP10	GUSBL2	H2AFV	HCG4P6	HDAC5	HIF1AN
HIGD1A	HIGD2B	HIST1H1E	HIST1H2AD	HIST1H2AG	HIST1H2BE
HIST1H2BF	HIST1H2BL	HIST1H3D	HIST1H3H	HIST1H4E	HMBOX1
HMGB2	HMMR	HN1	HNRNPL	HOXC8	HSP90AB1
HYLS1	ICA1L	IDE	IFITM1	IFT122	IFT88
IGSF8	IKZF2	IKZF5	ING4	INTS9	IRS1
ISG15	JARID2	KBTBD3	KCTD20	KCTD7	KDSR
KIAA0562	KIAA0753	KIAA1012	KIAA1522	KIAA1712	KIAA1715
KIAA1826	KIFC1	KPNB1	KRTAP20-3	LCA5	LDLR
LENG9	LEPR	LIMD1	LIN54	LOC100190939	LOC100294362
LOC143666	LOC256880	LOC284900	LOC348926	LOC552889	LOC646851
LOC728407	LOC729384	LPGAT1	LRIG2	M6PR	MALT1
MALT1	MAP3K3	MAP3K5	MAP3K7	MAPK1	MAPK15
MAPK7	MAPKAPK3	MBD4	MBLAC1	MCCC2	MCM10
MCM3	MED15	MED19	MED28	MED31	METT10D
METTL11A	METTL14	METTL2B	MIA3	MIF	MIR760
MLF1IP	MLPH	MOGS	MOSPD3	MPHOSPH8	MRAS
MRPL20	MRPL37	MRPL54	MRPS10	MRPS14	MRPS14
MRPS18B	MS4A6E	MSX2	MT2A	MTERF	MTMR7
MTRF1	MUL1	MYADM	MYCL1	MYL6	MYO1B
N4BP2	NAA20	NAA38	NACA	NAP1L1	NAT9
NBAS	NCOA5	NCRNA00171	NDUFB4	NDUFS5	NEAT1
NFIL3	NFKBIL1	NGDN	NHEDC1	NIF3L1	NIF3L1
NIPBL	NOL12	NPAS3	NRBF2	NRG4	NT5C
NTS	NUDCD2	NUDCD3	NUDT12	NUDT15	NUP35
OCIAD1	OPN5	OR10S1	OR12D2	OR12D3	OR4A16
OR51S1	OR52B4	OR5B12	OR5B3	OR5L2	OR5M1
OR8J1	ORMDL2	OSGIN2	OXGR1	P4HA2	PAPD4
PARG	PARP4	PCDHB16	PCOTH	PDGFRA	PEX10
PEX11G	PFKFB2	PGAM1	PGR	PHACTR4	PHF10
PHIP	PI4K2B	PIGU	PIK3R4	PLEKHG3	PMF1
PMVK	PNRC2	POLD1	POLD3	POLDIP3	POU2F1
PPAP2A	PPAP2A	PPP1R10	PPP1R15B	PPP2R3A	PRICKLE4
PRKCG	PRR11	PRR3	PSMB2	PTCHD2	PTGER1
PTGER3	PTGR2	PUSL1	PVRL3	PXT1	RAB36
RAB6A	RAD51AP2	RAD51L1	RAP1GAP	RASSF3	RB1
RBM33	RBM39	RBM47	RBP7	RGL2	RHOA
RHOT1	RIMKLA	RIMS3	RNASE4	RNF138P1	RNF150
RNF44	RNLS	RNMT	RNU11	ROBO2	RPA1

RPAP2	RPL13A	RPL13AP5	RPL18A	RPL18AP3	RPL41
RPN2	RPP14	RPS26	RPS7	RPS8	RPSA
RRBP1	RSPH4A	S1PR1	SCN8A	SDAD1	SDHD
SEC1	SEC11C	SELO	SEMA4B	SERINC4	SERINC5
SFPQ	SFRP4	SFRS11	SFRS2IP	SH3BP4	SH3BP5
SIRT7	SKA2	SLC15A4	SLC20A2	SLC30A5	SLC33A1
SLC3A2	SLC48A1	SLC8A3	SLIT1	SLU7	SMAGP
SMEK2	SMTNL2	SMYD4	SNORA6	SNORD26	SNORD27
SNORD28	SNORD29	SNORD46	SNORD55	SNX10	SNX19
SNX3	SNX8	SPG7	SPOP	SPTY2D1	SRBD1
SRCAP	SRM	SSBP4	SSH3	ST8SIA1	STOX2
STX5	STX6	SVIP	SYT17	TAF5	TANC1
TAOK3	TAX1BP1	TBC1D1	TCF3	TCF7L2	TCP11L2
TES	TFPI2	TGFBR2	THG1L	THNSL1	THOC5
TIMM8B	TLE6	TLR5	TLR6	TMEM104	TMEM159
TMEM19	TMEM205	TMEM223	TMEM52	TMEM56	TMEM8A
TMTC3	TMX2	TNFRSF11A	TOMM7	TOP3B	TPT1
TRIM24	TRIM5	TRMU	TSKU	TSPAN17	TSPYL1
TSSC4	TTC18	TTC37	TTLL6	TUBGCP2	TWF1
TWSG1	TXN2	TXNDC12	TXNDC17	TXNL1	UBA2
UBAP2L	UBE3A	UBN1	UCHL3	UNC13D	UNG
UQCRFS1	USP39	USP6NL	UTP20	VASP	VPS25
VPS35	VTRNA1-2	WAPAL	WDTC1	WDYHV1	XRCC2
XRCC4	YPEL2	ZBTB48	ZBTB7A	ZFAND6	ZFP91
ZKSCAN5	ZMYM6	ZNF137	ZNF140	ZNF17	ZNF195
ZNF215	ZNF227	ZNF333	ZNF398	ZNF425	ZNF507
ZNF514	ZNF519	ZNF557	ZNF562	ZNF600	ZNF643
ZNF664	ZNF668	ZNF680	ZNF695	ZNF703	ZNRD1
ZSWIM2	ZSWIM5	ZZZ3	IKBIP	LEPROT	TTC21A

**BOTH PROMOTER AND GENE BODY**					
BLOC1S2	C11orf73	C20orf132	C2orf60	C6orf217	CASC3
CLK1	DCTN4	ECD	GNL1	GPC5	KCNQ1OT1
LOC729991-MEF2B	LYSMD2	MCFD2	MIPEP	MLF2	MYO1E
NEDD9	NME2	PBX3	PDE4C	PDLIM5	PEX19
PSMA1	RCOR3	RGMB	SP3	TBC1D14	TMCO3
TMEM138	TNRC6B	TUBA4A	UGP2	USP2	ANGPT1
**UTR**					
ANGPT1	BAD	BEND6	C17orf99	C1orf187	DGKH
GABRA44	SHOX2	STATH	UPK3B	ZNF470	
**PROMOTER AND UTR**					
TAPBP	CASP4				
