# Supplementary material for: Genome-Wide Profiling of PARP1 Reveals an Interplay with Gene Regulatory Regions and DNA Methylation
Source: PLoS One. 2015 Aug 25;10(8):e0135410. doi: 10.1371/journal.pone.0135410 (PMC4549251; doi:10.1371/journal.pone.0135410)
Supplement: S3 Table — (PDF) [file pone.0135410.s013.pdf]

Table S3: HYPOMETHYLATED GENES AND GENOMIC REGIONS MEDIATED BY PARYLATION INHIBITION

**GENE BODY**					
AASDHPPT	ACADVL	ALK	BAT5	C1orf107	C7orf50
CAMTA1	CCDC64B	CKS1B	CREG1	FAM120AOS	FGF12
FTO	GABRB3	GNL1	GPR125	GPR98	HES2
HSD17B7P2	KIAA0319	KIAA1609	LOC285456	LOC439994	LRTOMT
MATN1	MED20	MED8	MINPP1	MOV10	MTHFS
MTX1	MYB	NAA38	NCRNA00171	NFIC	NOX4
NTM	PAR5	PCDHA1	PCDHA2	PCDHA3	PCDHA4
PCDHA5	PER4	PEX14	PLXNA2	PMS2L1	PTHLH
RBM22	RING1	RPS18	SERINC2	SHFM1	SLC25A46
SNORD64	SNRPC	SPDYA	SRRM3	SSH3	SUZ12P
TCERG1L	TMEM132D	TMEM41B	TPD52	TRIM27	TRIM69
TRIM77	TTC4	VIM	YME1L1		
**PROMOTER**					
ABHD13	ADAT2	ADD3	ADSL	ALDOC	AMOTL1
AP2B1	ARHGAP1	ARNTL	ART5	B4GALNT1	B9D2
BACH1	BCAR3	BCO2	BIRC3	BMPR1A	BYSL
C10orf131	C10orf71	C11orf31	C11orf46	C11orf59	C11orf83
C13orf36	C18orf10	C18orf55	C19orf47	C19orf56	C1orf84
C2orf69	C5orf30	C7orf64	CA8	CACNA1D	CACNA1G
CCDC109A	CCL11	CD164L2	CDC23	CDC42EP3	CDC45L
CHST11	CHTF18	CLDN22	CNST	COL4A3BP	COX16
CPNE5	CRYM	CSTF3	DCPS	DCTD	DDHD2
DERL2	DLG4	DLGAP2	DNAJC8	DPH3	DYNC1I2
EDC3	EFNA4	EIF2B1	EIF4A2	EPRS	ERBB2
ERLIN2	ERO1L	ESR1	FAM111A	FAM120A	FAM82A2
FAM86A	FANCF	FANCF	FARP2	FASTKD5	FBXO15
FGF14	FGFBP3	FLJ10661	FLJ32065	FOXJ3	GABRA4
GAK	GLYCTK	GPC6	GPD1L	GPD2	GPER
GPR157	GSTCD	GTF2H3	GTPBP10	HIF1AN	HINT3
HIST1H2AC	HIST1H2BF	HIST1H3D	HLA-C	HOMEZ	HSD17B12
INTS1	IQUB	ITCH	ITSN1_ITSN1_CRYZL1	JAZF1	KCNJ8
KIF2C	KLC4	KLKB1	KTN1	LEPROT	LHX1
LIG4	LIPT1	LMBRD2	LOC100286844	LOC100286844	LOC143666
LOC728190	MAP9	MASTL	MIS12	MOSC2	MRPL2
MRRF	MTERFD3	MTIF3	NAF1	NANP	NBR1
NCKAP5L	NCRNA00169	NDUFA11	NEIL2	NEU1	NHEDC1
NOL9	NUMB	NUS1	OR2G3	OR4A15	OR51A2
OR6P1	OR8H1	OSCP1	OXNAD1	PAFAH1B3	PAIP2
PAK1IP1	PARP1	PCBP2	PCDHA6	PCF11	PDE8A
PDLIM5	PER3	PEX1	PEX3	PHRF1	PIGK
PIK3C3	PIK3R2	PILRB	PLA2G12A	PLA2G6	PLD3
PMM1	POLK	POLR3A	PPP1R10	PPP1R3C	PRIC285
PRKAA2	PRR3	PTMS	PYHIN1	RAB30	RAB39
RALGAPA1	RARA	RARA	RBBP8	RBM18	RFWD3
RIC8B	RNF219	RPGRIP1L	RPL34	RPUSD1	RUFY1

RWDD1	SAP130	SCLY	SCRN2	SEC24D	SEMA5A
SERPINB4	SF3A3	SHC1	SKP2	SLC16A13	SLC22A23
SLC22A5	SLC2A1	SMAD4	SNRNP40	SPATA13	SRRT
STIM2	SUPT5H	SVIP	TAS1R1	TBC1D15	TBL1XR1
TFB2M	THBS1	THBS3	TIMM17A	TIMM22	TLE1
TMEM175	TMEM91	TNFRSF19	TRAM1L1	TRPS1	TSGA10
TUBA1B	UBL5	UBOX5	UFD1L	UGP2	USP48
VPS52	WDR19	YARS2	ZC3H13	ZCCHC17	ZNF230
ZNF251	ZNF259	ZNF317	ZNF350	ZNF408	ZNF684
ZNF697	ZNF74	ZNRD1	ZZZ3		
**PROMOTER AND GENE BODY**					
SEPT9	C6orf52	FNDC3A	KIAA1328	MOCS2	MORG1
**PROMOTER AND UTR**					
NLE1	PDE4C	PRDM2	TNFAIP8	ZNF323	CARD18
HIST1H2AD	LGI4	NPR3	PSMG4		
