# Supplementary material for: Genome-Wide Profiling of PARP1 Reveals an Interplay with Gene Regulatory Regions and DNA Methylation
Source: PLoS One. 2015 Aug 25;10(8):e0135410. doi: 10.1371/journal.pone.0135410 (PMC4549251; doi:10.1371/journal.pone.0135410)
Supplement: S4 Table — (PDF) [file pone.0135410.s014.pdf]

Table S4: Pathways of gene targeted by PARylation-mediated methylation

Hypomethylation:

A. Pathway commons analyses	
PathwayName	Statistics
Gene Expression	P = 5.41e-06
Developmental Biology	P = 9.49e-05
NOTCH	P = 0.0003
Notch signaling pathway	P = 0.0003
Notch-mediated HES/HEY network	P = 0.0003
mRNA Processing	P = 0.0009
Axon guidance	P = 0.0013
Formation and Maturation of mRNA Transcript	P = 0.0022
ErbB4 signaling events	P = 0.0026
mRNA Splicing - Major Pathway	P = 0.0027

B. KEGG pathway analyses	
PathwayName	Statistics
Metabolic pathways	P = 0.0021
Pathways in cancer	P = 0.0043
Phagosome	P = 0.0229
Glycerophospholipid metabolism	P = 0.0275
Focal adhesion	P = 0.0275
Ether lipid metabolism	P = 0.0275
TGF-beta signaling pathway	P = 0.0275
Small cell lung cancer	P = 0.0275
Ubiquitin mediated proteolysis	P = 0.0275
Spliceosome	P = 0.0275

Hypermethylated:

A. Pathway commons analyses	
PathwayName	Statistics
LKB1 signaling events	P = 1.79e-09
Signaling events mediated by focal adhesion kinase	P = 1.79e-09
VEGF and VEGFR signaling network	P = 1.79e-09
TRAIL signaling pathway	P = 1.79e-09
ErbB receptor signaling network	P = 1.79e-09
Arf6 trafficking events	P = 1.79e-09
Urokinase-type plasminogen activator (uPA) and uPAR-mediated signaling	P = 1.79e-09
Signaling events mediated by Hepatocyte Growth Factor Receptor (c-Met)	P = 1.79e-09
Sphingosine 1-phosphate (S1P) pathway	P = 1.79e-09
mTOR signaling pathway	P = 1.79e-09

B. KEGG pathways	Statistics
Metabolic pathways	P = 0.0001
Adherens junction	P = 0.0004
MAPK signaling pathway	P = 0.001
Protein processing in endoplasmic reticulum	P = 0.001
Ribosome	P = 0.001
Nucleotide excision repair	P = 0.0017
Homologous recombination	P = 0.0017
Systemic lupus erythematosus	P = 0.0017
Endocytosis	P = 0.003
Fc gamma R-mediated phagocytosis	P = 0.0037
