# Supplementary material for: Genome-Wide Profiling of PARP1 Reveals an Interplay with Gene Regulatory Regions and DNA Methylation
Source: PLoS One. 2015 Aug 25;10(8):e0135410. doi: 10.1371/journal.pone.0135410 (PMC4549251; doi:10.1371/journal.pone.0135410)
Supplement: S5 Table — (PDF) [file pone.0135410.s015.pdf]

Table S5: GENES THAT ARE BOTH HYPER- AND HYPOMETHYLATED BY PARylation and the genomic regions affected.

CAMTA1	PAK1IP1	HIST1H2AD	LOC14366	ZZR3	NCRNA00171	UGP2
AASDHPPT	ADD3	PDE4C	NHEDC	LEPROT	DNAJC8	GABRA4
GABRB3	C6orf52	GTPBP10	PPP1R10	GNL	HIST1H2BF	ZNRD1
NAA38	CSTF3	HIF1AN	PRR3	PDLIM5	SVIP	HISTH3D
SSH3	EPRS					

Genes with regions undergoing hypo and hypermethylated in different genic regions.

	HYPOMETHYLATED REGION	HYPERMETHYLATED REGION
PAK1P1	Promoter	Gene body
AASDHPPT	Gene body	Promoter
C6orf52	Gene body	promoter
GABRB3	Gene body	Promoter
HIST1H2AD	3'UTR	Promoter
NAA38	Gene body	Promoter
NCRNA0071	Gene body	Promoter
SSH3	Gene body	Promoter
GNL	Promoter	Gene body
PDLIMS	Promoter	Gene body
UGP2	Promoter	Gene body
GABRA4	Promoter	3'UTR
